# Deletion of miPEP in adipocytes protects against obesity and insulin resistance by boosting muscle metabolism

**DOI:** 10.1016/j.molmet.2024.101983

**Published:** 2024-07-01

**Authors:** Alexis Diaz-Vegas, Kristen C. Cooke, Harry B. Cutler, Belinda Yau, Stewart W.C. Masson, Dylan Harney, Oliver K. Fuller, Meg Potter, Søren Madsen, Niamh R. Craw, Yiju Zhang, Cesar L. Moreno, Melkam A. Kebede, G. Gregory Neely, Jacqueline Stöckli, James G. Burchfield, David E. James

**Affiliations:** 1 School of Life and Environmental Sciences, University of Sydney, Camperdown, New South Wales, Australia; 2 Charles Perkins Centre, University of Sydney, Camperdown, New South Wales, Australia; 3 School of Medical Sciences, University of Sydney, Camperdown, New South Wales, Australia

**Keywords:** Mitochondria, Adipose tissue, Insulin resistance, Peptidases, Metabolism, Skeletal muscle

## Abstract

Mitochondria facilitate thousands of biochemical reactions, covering a broad spectrum of anabolic and catabolic processes. Here we demonstrate that the adipocyte mitochondrial proteome is markedly altered across multiple models of insulin resistance and reveal a consistent decrease in the level of the mitochondrial processing peptidase miPEP.

**Objective:**

To determine the role of miPEP in insulin resistance.

**Methods:**

To experimentally test this observation, we generated adipocyte-specific miPEP knockout mice to interrogate its role in the aetiology of insulin resistance.

**Results:**

We observed a strong phenotype characterised by enhanced insulin sensitivity and reduced adiposity, despite normal food intake and physical activity. Strikingly, these phenotypes vanished when mice were housed at thermoneutrality, suggesting that metabolic protection conferred by miPEP deletion hinges upon a thermoregulatory process. Tissue specific analysis of miPEP deficient mice revealed an increment in muscle metabolism, and upregulation of the protein FBP2 that is involved in ATP hydrolysis in the gluconeogenic pathway.

**Conclusion:**

These findings suggest that miPEP deletion initiates a compensatory increase in skeletal muscle metabolism acting as a protective mechanism against diet-induced obesity and insulin resistance.

## Highlights

• Insulin resistance is characterised by a change in mitochondrial proteostasis in adipocytes.
• The mitochondrial peptidase miPEP is consistently downregulated in insulin resistance.
• Adipocyte-specific miPEP deletion confers protection against diet-induced obesity and insulin resistance.
• Deletion of miPEP in adipocytes increases muscle metabolism shielding against metabolic dysfunction induced by diet.

## Graphical abstract

**Figure:**
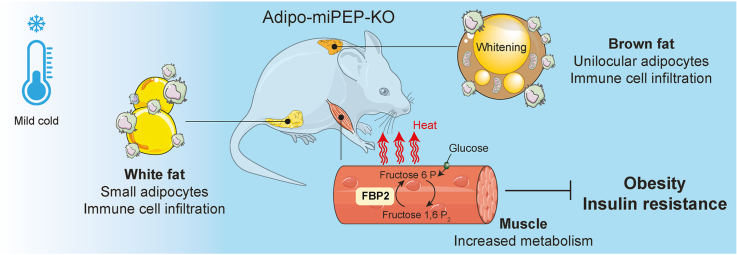

## 1 Introduction

Since their incorporation into ancestral cells ∼1.5 billion years ago, mitochondria have evolved into indispensable organelles within most eukaryotic cells [1]. They facilitate over 1,000 biochemical reactions, encompassing a diverse range of anabolic and catabolic functions beyond mere ATP generation [2]. These functions are heavily reliant on the mitochondrial proteome, which is dynamically regulated to adapt to changing environmental conditions [3], and loss of mitochondrial proteostasis profoundly impacts cellular homoeostasis and survival [3]. For example, loss of mitochondrial proteostasis is strongly associated with ageing and multiple diseases, including cardiometabolic diseases such as heart failure [4], type 2 diabetes [5] and cancer [6].

Insulin resistance, characterised by a deficiency in insulin-mediated glucose transport into muscle and fat tissues, is one of the earliest indicators of cardiometabolic disorders [7]. It typically coincides with a complex interplay of metabolic abnormalities, including obesity, hyperinsulinemia, hyperglycemia, and hyperlipidaemia, and is influenced by both genetic and environmental factors [[7], [8], [9]]. We and others have shown that insulin resistance leads to alterations in the mitochondrial proteins, independent of changes in gene expression, suggesting a potential role for mitochondrial protein stability in insulin resistance [[10], [11], [12], [13]]. For example, analysis of the mitochondrial proteome in human muscle biopsies has revealed a specific reduction in mitochondrial respiratory complexes I and III in insulin-resistant muscle, a finding consistent with observations in both animal and *in vitro* studies [10]. Moreover, insulin resistance has been associated with a remodelling of the mitochondrial proteome in adipose tissue [11,14]. This remodelling is characterised by a downregulation of several mitochondrial proteins leading to mitochondrial oxidative stress which plays a causal role in the pathogenesis of insulin resistance [10,11,15,16].

Mitochondria are naturally vulnerable to biochemical stresses [17], requiring intricate quality control mechanisms to repair or eliminate damaged mitochondrial proteins [18]. This surveillance system involves a network of evolutionarily conserved mitochondrial nuclear-encoded proteases and chaperones that are strategically distributed across various mitochondrial compartments [18]. Therefore, it is conceivable that the increased number of biochemical reactions due to nutrient oversupply could disrupt mitochondrial protein stability by overwhelming this surveillance system and thus lead to insulin resistance [19]. However, the specific mechanisms that cause changes in the mitochondrial proteome and how these changes relate to insulin resistance remain unclear.

In this study, we analysed publicly available datasets [11,14] to assess the impact of insulin resistance on the mitochondrial proteome in adipocytes. This analysis revealed a consistent decrease in the abundance of mitochondrial processing peptidase miPEP. To gain a deeper understanding of the potential role of this peptidase in insulin resistance, we developed and characterised the first adipocyte-specific miPEP knockout mouse model (adipo-miPEP-KO). Strikingly, and in contrast to our predictions, adipo-miPEP-KO mice were protected against diet-induced insulin resistance and obesity. We posit that this is compensatory increases in skeletal muscle metabolism serving as a protective mechanism against diet-induced metabolic dysfunction.

## 2 Materials and methods

### 2.1 Animals

MiPEP^fl/f^ mice were produced by the Mouse Engineering Garvan/ABR (MEGA) Facility (Moss Vale and Sydney, Australia) using CRISPR/Cas9 gene targeting in C57BL/6J mouse embryos following established molecular and animal husbandry techniques [20]. Two single guide RNAs (sgRNAs) were employed that targeted Cas9 cleavage 504 bp 5′ of Exon 2 and 275 bp 3′ of Exon 3 of Mipep (TGAGTGCCTCACATACA∗GCGAGG and ATTTGCTGTCAACAGTA∗CGGAGG respectively, ∗ = Cas9 cleavage site, protospacer-associated motif = PAM underlined). A homologous recombination (HR) substrate was synthesised in pUC57 plasmid (Genscript, Piscataway, NJ). This included a 3,318 bp insert corresponding to miPEP sequences from 1,260 bp 5′ of Exon 2 to 1,042 bp 3′ of Exon 3, with 34 bp loxP sequences inserted at the two Cas9 cleavage sites. A solution consisting of the two sgRNAs (15 ng/μL each), purified double stranded HR template plasmid DNA (2 ng/μL) and full length, polyadenylated S.pyogenes Cas9 mRNA (30 ng/μL) was prepared and microinjected into the nucleus and cytoplasm of C57BL/6J zygotes. Microinjected embryos were cultured overnight and those that underwent cleavage introduced into pseudo-pregnant foster mothers. Pups were screened by PCR to detect homologous recombination of the two loxP sites into one of the Mipep alleles. A founder mouse was backcrossed to wild-type C57BL/6J mice and progeny inter-crossed to derive the homozygous miPEP^fl/f^ line.

Genotyping sequencing was performed using the following primers: Cre (TG:790bp; WT: No product): Forward: 5′ CCGGTCGATGCAACGAGTGAT 3′; Reverse: 5′ ACCAGAGTCATCCTTAGCGCC 3′. miPEP (TG: 124bp; WT: 90 bp): Forward 5′ ACGTCTCCTTGTGGGTTATTGGTA 3′; Reverse: 5′ TAAAGCAGACAGTATGTATGAGT 3′. PCR reaction was performed by using PCR enzymes – Taq DNA Polymerase (MCLab, Molecular-cloning Laboratories). Genotyping via High resolution melt-curve analysis. Initial Denaturation was at 94 °C for 10 s, 10 cycles at 94 °C for 10 s, 65–55 °C for 30 s, 60 °C for 1 min/kb followed by 30 circles at 94 °C for 10 s, 55 °C for 30 s and 60 °C for 1 min/kb and a final extension step at 72 °C for 3 min.

All animal experiments were carried out in accordance with the NHMRC (Australia) guidelines for animal research and were approved by the University of Sydney Animal Ethics Committee. miPEP^flox/flox^ mice were bred with miPEP^flox/flox^/Adiponectin-Cre^+/−^ mice to generate all adipo-miPEP-KO and miPEP^flox/flox^ littermates used for experiments in this study. Mice were group-housed on a 12 h light/dark cycle with free access to food and water.

### 2.2 Isolation of mature adipocytes

Adipocytes were isolated from gonadal white adipose tissue (gWAT) and subcutaneous adipose tissue (SAT) following a previously described protocol with minor modifications [21,22]. Adipose tissue was minced in fresh buffer (120 mM NaCl, 4.7 mM KCl, 1.18 mM KH_2_PO_4_, 1.17 mM MgSO_4_·7H_2_O, 2 mM CaCl_2_, 30 mM HEPES, 10 mM NaHCO_3_, 5 mM glucose, 1% BSA, pH 7.4) until pieces were smaller than 1 mm^2^. For digestion, type I collagenase (Worthington, Cat#LS004194) was added at a concentration of 0.5 mg/mL for gWAT and 1 mg/mL for SAT. The samples were then placed in a shaking water bath at 100 rpm for 1 h at 37 °C. After digestion, the samples were filtered through a 250 μm or 300 μm nylon mesh (Spectrum Labs) for chow- and high-fat diet-fed mice, respectively. The isolated adipocytes were washed three times with HES buffer (250 mM sucrose, 20 mM HEPES, 1 mM EDTA, pH 7.4). Between washes, adipocytes were left for 5 min to form a floating layer, and the infranatant was removed by aspiration using a Hamilton syringe.

### 2.3 Immunoblotting

To assess miPEP abundance in isolated adipocytes, the cells were lysed in 2 % SDS lysis buffer supplemented with protease and phosphatase inhibitors (Thermo Fisher Scientific, Cat#A32965). Protein concentration was determined using a BCA assay (Thermo Fisher Scientific, Cat#A558600). The lysates, along with Novex pre-stained molecular weight markers (Thermo Fisher Scientific, Cat#26620), were loaded onto SDS-PAGE gels and transferred to PVDF membranes. Immunoblotting was performed using primary antibodies (anti-miPEP, Santa Cruz Biotechnology, San Francisco, CA, USA, Cat#ab154407) and either infrared dye 700- or 800-conjugated secondary antibodies (Thermo Fisher Scientific, Cat#A32735 & A-21036). Detection was carried out using an Odyssey CLx system (Li-Cor). Densitometry analysis, normalised to the loading control (14-3-3, Santa Cruz, sc-629, clone K19, Cat#ab2273154), was conducted using ImageStudioLite software (Li-Cor).

### 2.4 Physiological assays

The body composition of mice was determined using Echo-MRI. Glucose tolerance tests (GTT) and insulin tolerance tests (ITT) were conducted on both male and female mice following a 6-h fast. For the GTT, a bolus of glucose (2 g/kg lean mass) was administered via oral gavage. Blood glucose levels were measured every 15 min monitored during the GTT to calculate the Area of the curve (AOC). This was calculated by measuring the area under the curve of the GGT and subtracting the area under the baseline as described [23]. Insulin (1 U/kg lean mass) was injected intraperitoneally for the ITT. Prior to insulin injections, the tail was snipped 10 min before the experiment. Blood glucose and insulin levels were measured at indicated time points by sampling blood from the tail tip using an Accu-Check II glucometer (Roche Diagnostics) or an insulin ELISA kit (Crystal Chem Cat#90080), respectively. Plasma was obtained by collecting blood in EDTA-coated tubes (Sarstedt, Cat#41.1504.005) and centrifuging at 2000 ×*g* for 10 min at 4 °C. Plasma free fatty acids were measured using the NEFA C kit (Wako, Cat#279-75401). Plasma measurements before and after insulin injection utilised the 0 and 10-min plasma samples obtained during the ITT. This plasma was also used to measure FGF21 by ELISA (Abcam, Cat#ab212160). Islet isolation, total insulin content and glucose-stimulated insulin secretion assays were performed as previously described [24]. Triglycerides were extracted from approximately 30 mg of tissue using a 2:1 chloroform:methanol mixture, as previously described [25]. The liquid was evaporated using a GeneVac concentrator, and lipids were resuspended in isopropanol. Triglycerides were measured using a triglyceride kit (Thermo Fisher Scientific, Cat#EEA028) and normalised to tissue weight. For lipid tolerance tests mice were fasted overnight followed by an oral gavage of intralipid (15 μL/g body weight) (Sigma, Cat#I141). Blood was collected from the tail (∼20 μL) every hour for 6 h. Samples centrifuged at 2000 ×*g* for 10 min at 4 °C and triglycerides were measured using a triglyceride kit (Thermo Fisher Scientific, Cat#EEA028) as previously described [26].

### 2.5 Metabolic cage studies

Metabolic cage studies were conducted at the Charles Perkins Centre, University of Sydney. Mice were maintained on a 12-h dark–light cycle at room temperature. Prior to each experiment, mice were acclimated in the metabolic chambers for 2 days. Metabolic parameters, including oxygen consumption, CO2 generation, food intake, and water consumption, were continuously monitored and recorded using the Metabolic cage system (Promethion, Sable Systems, Las Vegas, NV, USA). Throughout the study, all transgenic mice and their control miPEP^fl/fl^ littermates were individually housed and provided with *ad libitum* access to normal chow or high fat high sugar diet and water.

### 2.6 Thermoneutrality exposure

At 13 wks old, both the adipo-miPEP-KO and miPEP^fl/fl^ littermates were housed in thermoneutrality conditions (30 °C, 50 % humidity, thermo-chamber), with free access to water and food. After one week of acclimatisation, the chow diet was substituted with a high-fat, high-sugar diet, and mice were maintained on a 12-h light/dark cycle with unrestricted access to food and water. These conditions were sustained for an additional four weeks.

### 2.7 Dual tracer test

The Dual Tracer Test (DTT) was performed as originally described [27]. Briefly, mice were fasted for 2 h between 1000 and 1200 and anaesthetised with sodium pentobarbital (65 mg/kg total body mass). Euthermia was maintained by wrapping anaesthetised mice in foil and placing them on heat pads. Mice were injected retro-orbitally with 2.5 μCi [14C]2DG and blood samples collected at 2-, 15- and 30-min post-injection. The second retro-orbital injection containing 5 μCi [3H]2DG and 0.75 U/kg lean mass insulin was performed at 40 min, and samples collected 2-, 15-, 30- and 40-min post injection. Mice were euthanized by cervical dislocation and tissues rapidly excised and snapped frozen in liquid nitrogen before storage at −80 °C. Systemic 2DG kinetics were quantified by using the slope of the exponential tracer disappearance curve. Tissue were ground in a liquid nitrogen cooled mortar and pestle and lysed in 1% Triton X-100 (Sigma–Aldrich, Cat#9002-93-1). Glucose uptake was normalised against tissue mass. Non-phosphorylated 2DG was isolated by running total tissue lysates through columns packed with AG 1-X8 anion exchange resin (BioRad, Cat#1401443). Tracers were detected by liquid scintillation counting and phosphorylated 2DG (2DG-6P) was calculated by subtracting non-phosphorylated counts from total counts. Tissue-specific tracer uptake rates were calculated using the method of Hom et al. [28] to adjust 2DG-6P accumulation for initial dose and changes in systemic availability of the tracer:

Where ki is the rate of uptake in tissue i, Ci is the amount of 2DG-6P in the tissue at the end of the experiment, kc is the rate of tracer disappearance from circulation, d0 is the initial concentration of tracer in circulation extrapolated from a single exponential curve and t is the duration that tissues are exposed to the tracer (t = 80 and 40 for basal and insulin-stimulated tracers, respectively). The effect of insulin was determined by calculating the area of the curve. This was done by measuring the area over the curve after insulin stimulation and then subtracting the area over the baseline.

### 2.8 Histological analysis

Tissues were fixed in 10 % neutralised formalin for 48 h, followed by three washed with 80 % ethanol. Samples were then sent to the Histology Facility of Garvan Institute (Sydney, Australia) for embedding, sectioning, and H&E or Picrosirius Red staining (PSR). All Images were obtained using Keyence BZ-X710 microscope. Analysis of adipose tissue fibrosis and adipocyte size were performed using ilastik [29] and custom macros in FIJI. Given that not every section underwent PSR staining, a subset of H&E-stained tissues with corresponding PSR stained sections used for reference were categorised into “fibrotic tissue”, “blood vessels”, “other tissues”, and “background”, utilising ilastik's pixel-based segmentation toolkit. Following training, the model was applied to H&E stained tissue sections for all samples. The percentage of fibrosis was quantified by comparing the area of “fibrotic tissue” to that of “other tissue”, excluding “blood vessels”. For adipocyte size assessment, H&E sections underwent processing with a custom FIJI macro that executed several steps: initial separation of tissue from the background through thresholding, removal of outliers smaller than 2 pixels, and determination of adipocyte area using FIJI's Analyse Particles function, with a size range set between 10 and 10,000 μm^2^ and a circularity range of 0.2–1.0. The distribution of adipocyte cell sizes was determined and then normalised to the total cell count per sample to give the proportion of cells in each size bin. Finally, the average of the bins for each group were calculated. For adipocyte size we used Chi-squared test.

### 2.9 Sample preparation for proteomics

Frozen adipose tissue was powdered and lysed with a dounce homogeniser in 4 % sodium deoxycholate (SDC, Sigma, Cat#S1827) and 100 mM tris–HCl (pH 8.5) at RT. Directly following lysis, samples were heated to 95 °C for 10 min with shaking using a thermomixer C (Eppendorf). Samples were allowed to cool to RT and sonicated for 30 s in 3 s on/off intervals at 90 % amplitude using a tip probe sonicator. Samples were centrifuged at RT for 10 min at 18,000 ×*g*. Lipid layer was aspirated and supernatant collected whilst avoiding any pelleted material.

For soleus, muscles were homogenised to powder using a liquid nitrogen cooled mortar and pestle. Samples were then lysed in 4 % SDC and 100 mM TRIS–HCL (pH 8.5) and boiled at 95 °C for 10 min with 2000 rpm shaking using a thermomixer C (Eppendorf). Samples were then sonicated for 30 s at 70 % amplitude using a tip probe sonicator before being centrifuged at RT for 10 min at 18,000 ×*g* to clarify lysate.

BCA total protein assay (Pierce, Cat#23225) was used according to manufacturer's instructions to determine protein concentration in samples. 20 μg of protein per lysate was concurrently reduced with 10 mM TCEP and alkylated with 40 mM chloroacetamide at 95 °C for 30 min. Samples were diluted with 100 mM Tris–HCl (pH 8.5) to a final SDC concentration of 1 % and digested for 16 h at 37 °C whilst shaking with MS-grade trypsin (Thermo Fisher Scientific, Cat#90057) and lysyl endopeptidase (Wako, Cat#25-02543) with each protease added at a ratio of 1:20 protease to protein.

Frozen plasma was defrosted on ice and 1 μL of plasma was added to 24 μL of SDC buffer (1 % SDC, 100 mM Tris–HCl pH 8.5, 40 mM chloroacetamide and 10 mM TCEP) and heated to 95 °C for 30 min with shaking to denature, reduce and alkylate samples. Once cooled, samples were diluted 10-fold with 100 mM Tris–HCl (pH 8.5) and digested as above.

In all tissues, SDC was precipitated from peptide samples by addition of 99 % ethyl acetate and 1 % trifluoroacetic acid (TFA, ChemSupply, Cat#TS181) (50 % final concentration, v/v) and vortexed until all SDC precipitate was resuspended. Sample cleanup was performed as previously reported [30]. Dried peptide samples were resuspended in 5 % formic acid and stored at 4 °C until LC–MS/MS analysis.

### 2.10 Mass spectrometry acquisition and analysis

Protein mass spectrometry was performed using either a ThermoFisher Orbitrap Exploris 480 or a ThermoFisher Q Exactive HF-X Quadrupole-Orbitrap utilising previously reported parameters except with a flow gradient reduced to 90 min [31]. RAW data was analysed using the DIA proteomics search engine, DIA-NN (version 1.8.1), as previously reported [31,32]. The output of the DIANN searches was uploaded to the ProteomeXchange Consortium under the identifier PXD051606.

### 2.11 Proteomics data analysis

Analysis of the adipose tissue and muscle proteome was performed with R (version 4.2.1). Data was filtered for proteins detected in at least 50 % of the samples, median normalised and log transformed. Identification of differentially regulated proteins between each group were performed using the R package limma [33] and p-values were corrected with p.adjust (method = “fdr”) within each comparison. Gene set enrichment was performed with the GSEA_4.3.2 as previously described [34].

### 2.12 Data availability

The proteomic datasets generated during this study have been deposited to the ProteomeXchange Consortium (http://www.proteomexchange.org/) via the PRIDE partner repository with the dataset identifiers PRIDE: PXD051606. **To access these data please use User_name:** reviewer_pxd051606@ebi.ac.uk, **Password: Fld3rfgR**.

### 2.13 Proteomics deconvolution

We employed the R package BisqueRNA [35] to perform reference-based decomposition of the entire tissue proteomics dataset, using parameters ‘marker = NULL’ and ‘use.overlap = FALSE’. For reference, we utilised murine single-cell RNA sequencing data [36] obtained in Seurat format, downloaded from https://gitlab.com/rosen-lab/white-adipose-atlas as we previously described [14].

### 2.14 Ex vivo islet isolation

Pancreatic islets were isolated as previously described [9] with injection of Liberase (Roche, Basel, CH) via the common bile duct. Pancreata were then processed in a dual Histopaque 1077 (Sigma–Aldrich, Cat#1119) gradient and handpicked under a stereomicroscope. Isolated islets were recovered in Islet Media (RPMI Thermofisher, Cat#12633020, 10 % FBS, 1 % penicillin/streptomycin) prior to glucose-stimulated secretion assay.

### 2.15 *Ex vivo* islet glucose-stimulated insulin secretion

Glucose-stimulated insulin secretion assays were performed as previously described [9]. Briefly, isolated islets were incubated for 1 h at 37 °C, 5 % CO_2_ in 2.8 mM glucose Krebs Ringer buffer supplemented with 10 mM HEPES (KRBH) (Thermofisher, Cat# 15630080) prior to stimulation at either 2.8 mM or 16.7 mM glucose in KRBH for 1 h at 37 °C, 5% CO_2_. Islet pellets were lysed with Islet Lysis Buffer (100 mM Tris, 300 mM NaCl, 10 mM NaF, 2 mM sodium orthovanadate). Insulin secretion in supernatant and total insulin content of islets were measured using an Ultra-sensitive insulin homogeneous time-resolved fluorescence assay (Cisbio, PerkinElmer, Cat#62IN2PEG).

### 2.16 Statistical analysis tissue proteome

Data are presented as mean ± S.D. with individual data points shown, unless otherwise indicated. Each data point is an independent biological experiment. Statistical analyses were performed in GraphPad Prism (version 10) or software R using two-tailed t-test (if not otherwise indicated) or one-way or two-way analysis of variance (ANOVA) with post-hoc analysis using Tukey's or Sidak's multiple comparison tests. Significance was set at p < 0.05 and p-values are indicated in the Figures.

## 3 Results

### 3.1 The mitochondrial peptidase miPEP is downregulated in insulin resistant adipocytes

**Figure 1:**
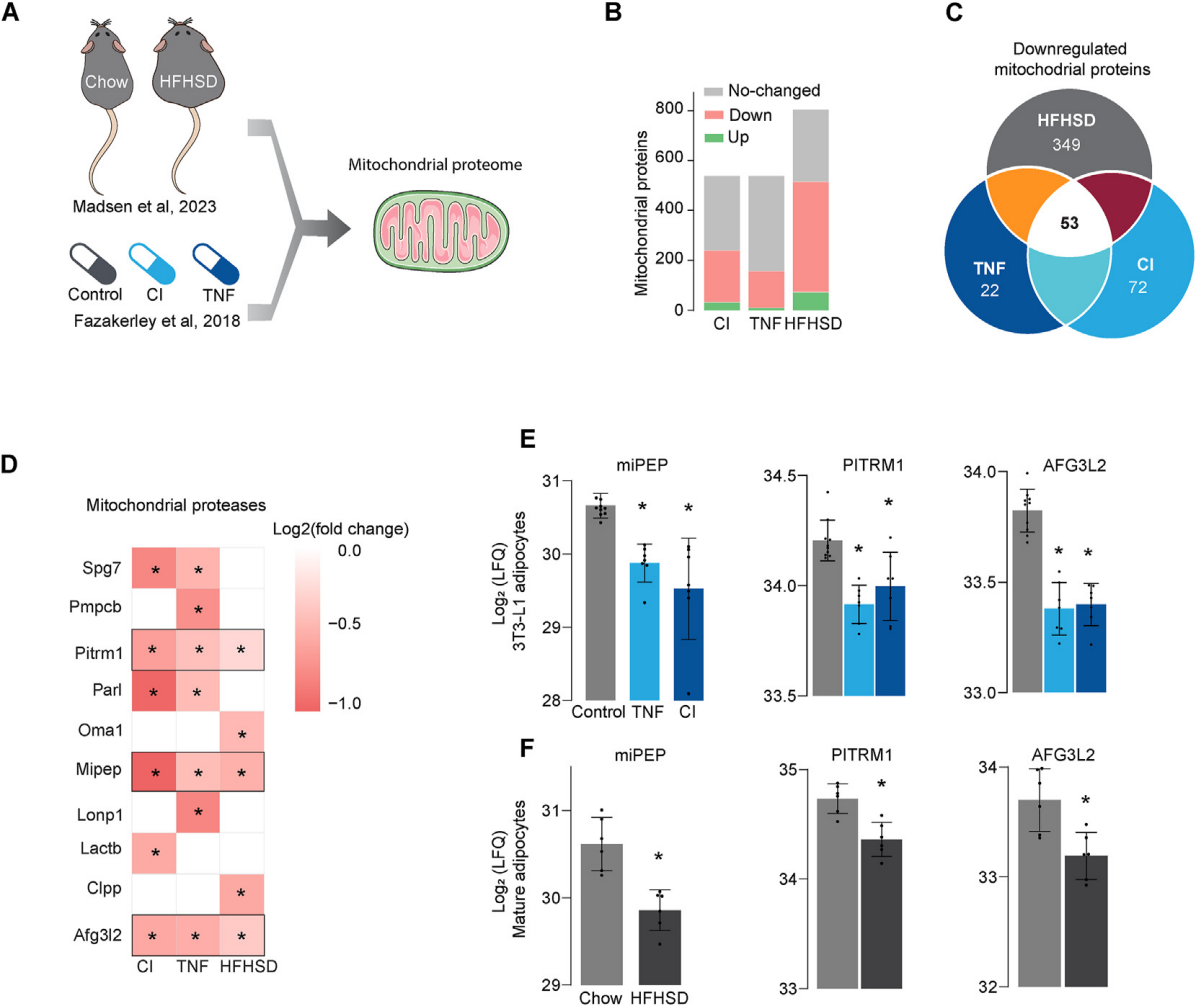
**The mitochondrial protease miPEP is downregulated in insulin resistant adipocytes** (**A**) Schematic of the analysis of two public available datasets [11,14]. (**B**) Overview of the mitochondrial proteome differentially regulated across insulin resistant models (Padj < 0.05). (**C**) Venn Diagram showing overlap between down regulated mitochondrial proteins across insulin resistant models. (**D**) Heatmap of canonical mitochondrial proteases proteins differentially regulated in at least one model of insulin resistanc (Padj < 0.05 and FC > −1.25). (**E**) Levels of mitochondrial proteases downregulated in insulin resistant 3T3-L1 adipocytes (**F**) and adipocytes isolated from mice exposed to high fat high sugar diet (HFHSD) or chow control N = 6–9 for 3T3-L1 adipocytes and 4–6 for mature adipocytes. Mean ± S.D. ∗p < 0.05 versus control. CI: Chronic insulin, TNF: Tumour Necrosis Factor ∝.

We utilised two publicly available datasets to identify changes in the mitochondrial proteome that may contribute to insulin resistance in adipocytes, one of the primary targets for insulin. These datasets included proteomic datasets of adipocytes isolated from mice exposed to a high-fat, high-sugar diet (HFHSD) [14] and 3T3-L1 adipocytes that had been subjected to different models of insulin resistance [11]. We integrated and filtered these datasets for the mitochondrial proteome using mitoCarta 3.0 [37] (Figure 1A). We identified ∼600 and ∼800 mitochondrial proteins in 3T3-L1 adipocytes and isolated mature adipocytes, accounting for 50%–70% of the total mitochondrial proteome, respectively.

In 3T3-L1 adipocytes, insulin resistance induced by chronic inflammation (chronic Tumour Necrosis Factor-alpha incubation, TNF) or hyperinsulinemia (chronic insulin incubation, CI) was sufficient to cause a marked downregulation of mitochondrial proteins. In primary adipocytes isolated from mice exposed to HFHSD, approximately 300 mitochondrial proteins were downregulated (Figure 1B), and only 53 proteins were commonly downregulated across all insulin resistance models (Figure 1C). Given the potential link between insulin resistance and mitochondrial proteostasis [10,11,14,15], we filtered the data for mitochondrial proteases. Several mitochondrial proteases were downregulated, albeit not consistently, across both *in vitro* and *in vivo* models. Specifically, 85% of the detected proteases were downregulated in at least one *in vitro* model, whereas only 35% of them exhibited downregulation in insulin resistant mature adipocytes (Figure 1D). Only the mitochondrial processing peptidase miPEP and the ATP-dependent proteases PIRTM1 and AFG3L2 were downregulated across all insulin resistance models (Figure 1D). While miPEP is crucial in maturation of newly imported mitochondrial proteins, Pirtm1 and Afg3l2 are quality control proteases that prevent the accumulation of immature mitochondrial proteins [18]. Among these mitochondrial proteases, miPEP exhibited the largest effect (Figure 1E,F) and was the only protease that was associated with metabolic diseases according to Human Genetic Evidence (HuGE) scoring [38], available in the Common Metabolic Diseases Knowledge Portal (https://hugeamp.org/). These data demonstrated that insulin resistance was associated with a change in the machinery responsible for mitochondrial proteostasis, leading to impaired protein maturation and the quality control, which may explain the variety of mitochondrial defects observed in insulin resistance [5,10,11,13,16].

### 3.2 Adipocyte specific miPEP deletion improves insulin sensitivity and lowers adiposity

**Figure 2:**
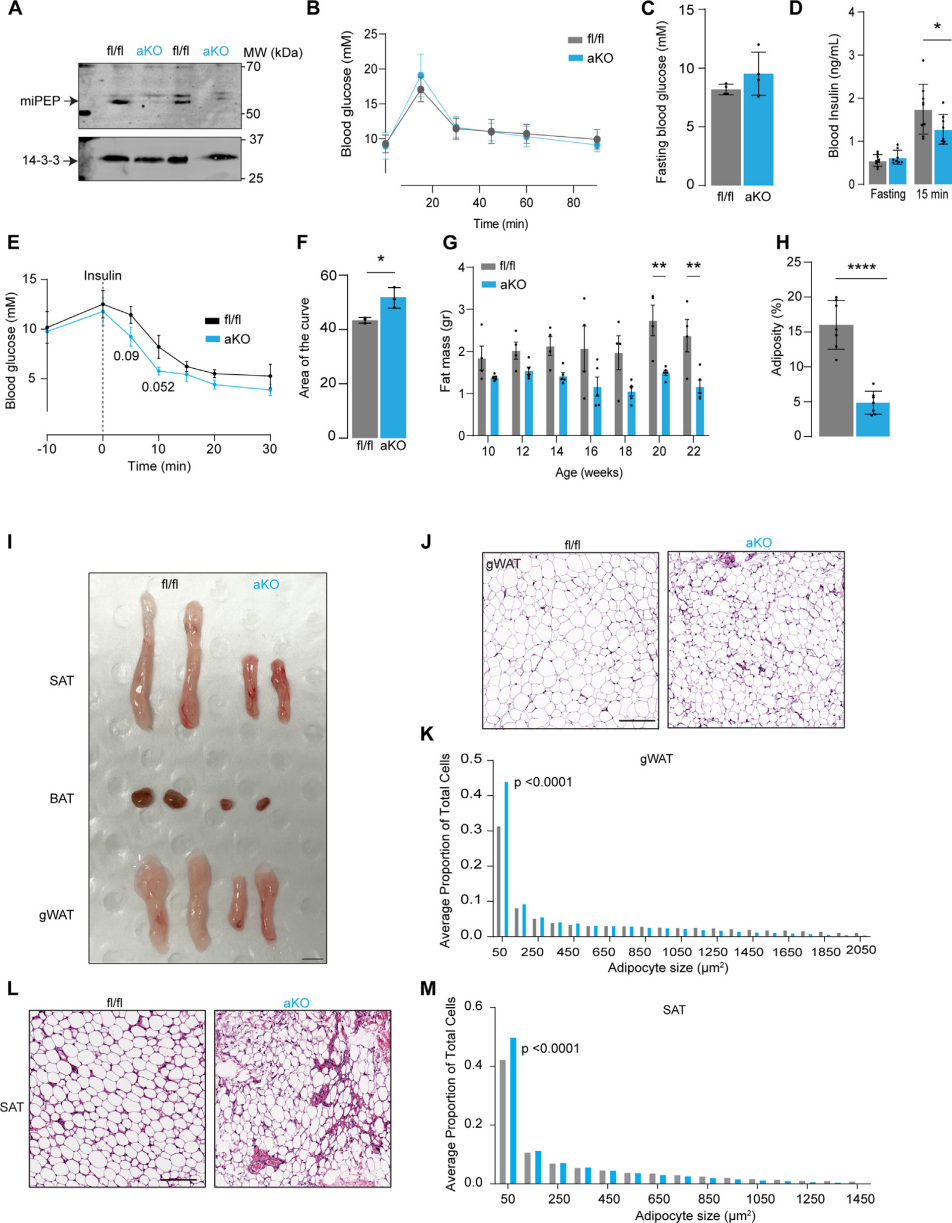
**Adipocyte specific miPEP deletion reduces adiposity and increases insulin sensitivity** (**A**) Western blotting against miPEP in control (miPEP^fl/fl^) or adipocyte specific KO mice (aKO) obtained from isolated adipocytes. (**B**) Oral Glucose tolerance test (**C**) Fasting blood glucose (**D**) Blood insulin (fasting and at 15 min during Oral Glucose tolerance test). Mean ± S.D., N = 7, ∗p < 0.05 vs miPEP^fl/fl^ mice. (**E**) Intraperitoneal insulin tolerance test (**F**) Area of the curve during insulin tolerance test. Mean ± S.D., N = 3, ∗p < 0.05 vs miPEP^fl/fl^ mice. (**G**) Temporal changes in fat mass (H) Adiposity at 22 wks old. N = 4–6, mean ± S.D. ∗∗p < 0.01, ∗∗∗∗p < 0.0001 vs miPEP^fl/fl^. (**I**) Representative image of adipose depots in miPEP^fl/fl^ and aKO mice. gWAT: Gonadal white adipose tissue, SAT: Subcutaneous white adipose tissue, BAT: Brown adipose tissue. Scale bar 1 cm. Haematoxylin and eosin staining of gWAT (**J**) and SAT (**L**) of miPEP^fl/fl^ and aKO mice. Scale bar 100 μm. Quantification of the adipocyte size distribution for gWAT (**K**) and SAT (**M**) from the H&E staining, N = 5, p-value reported.

Given that miPEP was significantly downregulated in insulin resistance, we next sort to evaluate the *in vivo* roles of miPEP in insulin resistance. To this end, we used a Cre/Lox system to create adipocyte specific miPEP knockout mice (adipo-miPEP-KO) and floxed miPEP control littermates (miPEP^fl/fl^). We observed almost complete absence (∼90%) of the miPEP protein in adipocytes isolated from white adipose tissue (Figure 2A). MiPEP deficient mice are overall healthy, exhibit normal postnatal life span or fertility (data not shown).

Since we predicted that miPEP might play a role in metabolic disease we next examined the metabolic phenotype of KO mice. While there was no difference in fasting glucose levels or glucose handling during an oral glucose tolerance test (OGTT) between adipo-miPEP-KO and miPEP^fl/fl^ mice (Figure 2B,C), miPEP deficient mice exhibited lower blood insulin at 15 min after oral glucose administration (Figure 2D). This suggests an increase in whole body insulin sensitivity in adipo-miPEP-KO mice. This was confirmed, as adipo-miPEP-KO mice were more insulin-sensitive during an insulin tolerance test (Figure 2E & F). Importantly, fasting blood glucose levels were measured 10 min before insulin administration to account for hyperglycemia associated with mouse handling (time: 10) (Figure 2E). This effect was not due to changes in β-cell function as *ex vivo* glucose-induced insulin secretion and insulin content was similar between the groups (Sup. Figure 1C,D).

Strikingly, unlike control mice adipo-miPEP-KO animals did not exhibit a time-dependent increase in fat mass when fed a normal chow diet (NCD) (Figure 2G). Adipo-miPEP-KO had 52% less adipose tissue at 20 wks old, with no difference in lean mass or body weight (Figure 2G, H, Sup. Figure 1C,D). Adiposity was reduced by 70.5% in adipo-miPEP-KO mice (Figure 2H). No difference in adiposity was observed between miPEP^fl/fl^ and age-matched wild type mice, indicating that the observed phenotype is driven by deletion of miPEP (Sup. Figure 1E). The decrease in adipose tissue mass in adipo-miPEP-KO was observed in all adipose depots including subcutaneous (SAT, ∼50% reduction), gonadal (gWAT, 65% reduction) and brown adipose tissue (BAT, 30% reduction) (Figure 2I, Sup. Figure 1F). H&E staining of gWAT (Figure 2J,K) and SAT (Figure 2L,M) showed a shift toward small adipocytes (50 μm^2^ in size) reducing the proportion of large adipocytes (over 1000 μm^2^ in size) in adipo-miPEP-KO mice (p < 0.0001 and p < 0.0001, for gWAT and SAT, respectively). A similar phenotype was observed in adipo-miPEP-KO female mice (Sup. Fig. 2). These findings indicate that miPEP deletion in adipocytes leads to reduced adiposity and this likely contributes to improved insulin sensitivity.

### 3.3 MiPEP-deficient mice are protected from diet-induced obesity and insulin resistance

**Figure 3:**
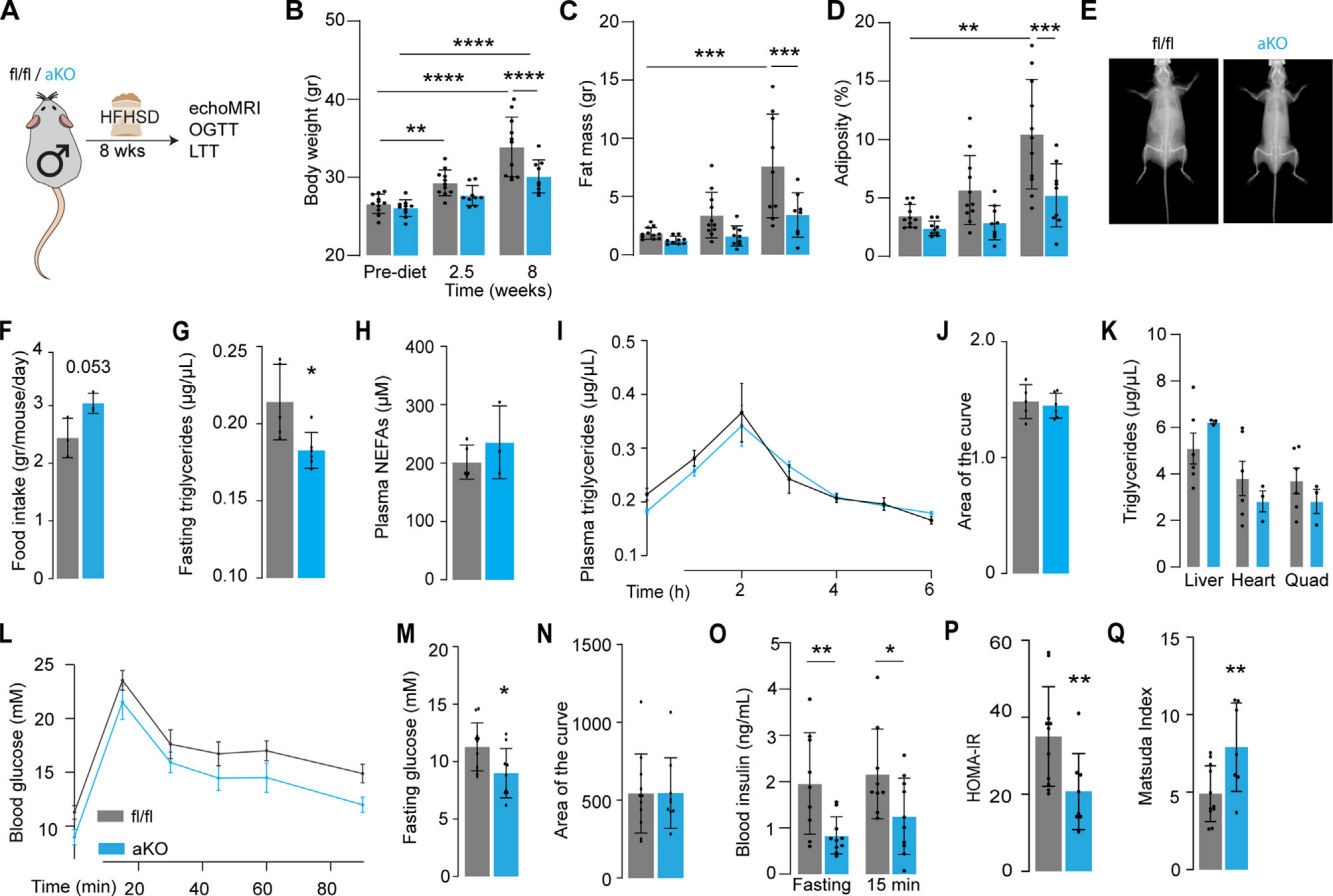
**miPEP deficient mice are protected against diet induced obesity and glucose intolerance**. (**A**) Experimental workflow HFHSD = High fat, high sugar diet. OGTT = Oral glucose tolerance test, LTT = Lipid Tolerance Test. Changes in (**B**) body weight, (**C**) fat mass and (**D**) adiposity pre-diet, at 2.5 wks and 8 wks of diet is shown. Mean ± S.D., N = 9–11, ∗∗p < 0.01, ∗∗∗p < 0.001, ∗∗∗∗p < 0.0001. (**E**) Representative dual energy X-ray absorptiometry (DEXA) image scan for control (miPEP^fl/fl^, fl/fl) and adipo-miPEP-KO (aKO) after 8 wks of diet intervention. (**F**) Food intake Mean ± S.D., p-value is reported. (**G**) Fasting triglycerides and (H) plasma NEFAs. Mean ± S.D., N = 5–6. ∗p < 0.05 vs miPEP^fl/fl^ mice. (**I)** Kinetic and (**J**) area of the curve of lipid tolerance test using oral gavage of intralipids. Mean ± S.D., N = 5–6 (K) Triglycerides levels across tissues in miPEP^fl/fl^ (fl/fl, grey) and adipo-miPEP-KO (aKO, light blue) mice. Mean ± S.D., N = 3–6. (**L**) Oral Glucose tolerance test, (**M**) Fasting glucose was determined after 8 wks of HFHSD. (N) Area of the curve was determined from the Oral Glucose tolerance test. (**O**) Fasting Blood insulin and at 15 min during the Oral Glucose tolerance test. (**P**) HOMA-IR (**Q**) Matsuda Index. Mean ± S.D., N = 8–10, ∗p < 0.05, ∗∗p < 0.01 vs miPEP^fl/fl^ mice.

Because of the well-established association between adiposity and insulin resistance [7] we hypothesised that miPEP deficient mice might be protected against insulin resistance. To test this, adipo-miPEP-KO and miPEP^fl/fl^ littermates were fed HFHSD *ad libitum* for 8 wks [24]. HFHSD-fed miPEP-deficient mice showed significantly lower body weight, fat mass and adiposity compared to flox mice, regardless of gender (Figure 3B–E, Sup. Fig. 2). This was not due to reduced food intake as if anything food intake was slightly elevated (16%) in adipo-miPEP-KO mice compared to miPEP^fl/fl^ mice (Figure 3F). Fasting non-esterified fatty acid (NEFA) levels were similar across genotypes, while fasting triglyceride levels were significantly lower (18%) in adipo-miPEP-KO mice (Figure 3G–H). To test whether adipo-miPEP-KO mice had impaired ability to absorb lipids, we performed a lipid tolerance test [26].We found that adipo-miPEP-KO mice exhibited comparable lipid absorption and triglyceride clearance compared to miPEP^fl/fl^ mice, suggesting that the lean phenotype is not due to defective nutrient absorption (Figure 3I–J). We did not observe differences in triglyceride levels in liver, heart, and quadriceps (Figure 3K). Furthermore, adipo-miPEP-KO mice showed similar glucose tolerance measures by the area of the curve during the GTT and improved insulin sensitivity evidenced by lower fasting blood glucose, and lower blood insulin levels (Figure 3M–P). In line with these results, HOMA-IR was 47% lower and the Matsuda index was 60% higher in male adipo-miPEP-KO mice fed a HFHSD (Figure 3P, Q). Similar results were observed across all these parameters in female adipo-miPEP-KO mice fed with HFHSD (Sup. Figure 2). Overall, these data suggest that miPEP deletion in adipocytes is sufficient to confer protection against diet-induced obesity and insulin resistance.

### 3.4 Thermoneutrality reverses the protective phenotype observed in adipo-miPEP deficient mice

**Figure 4:**
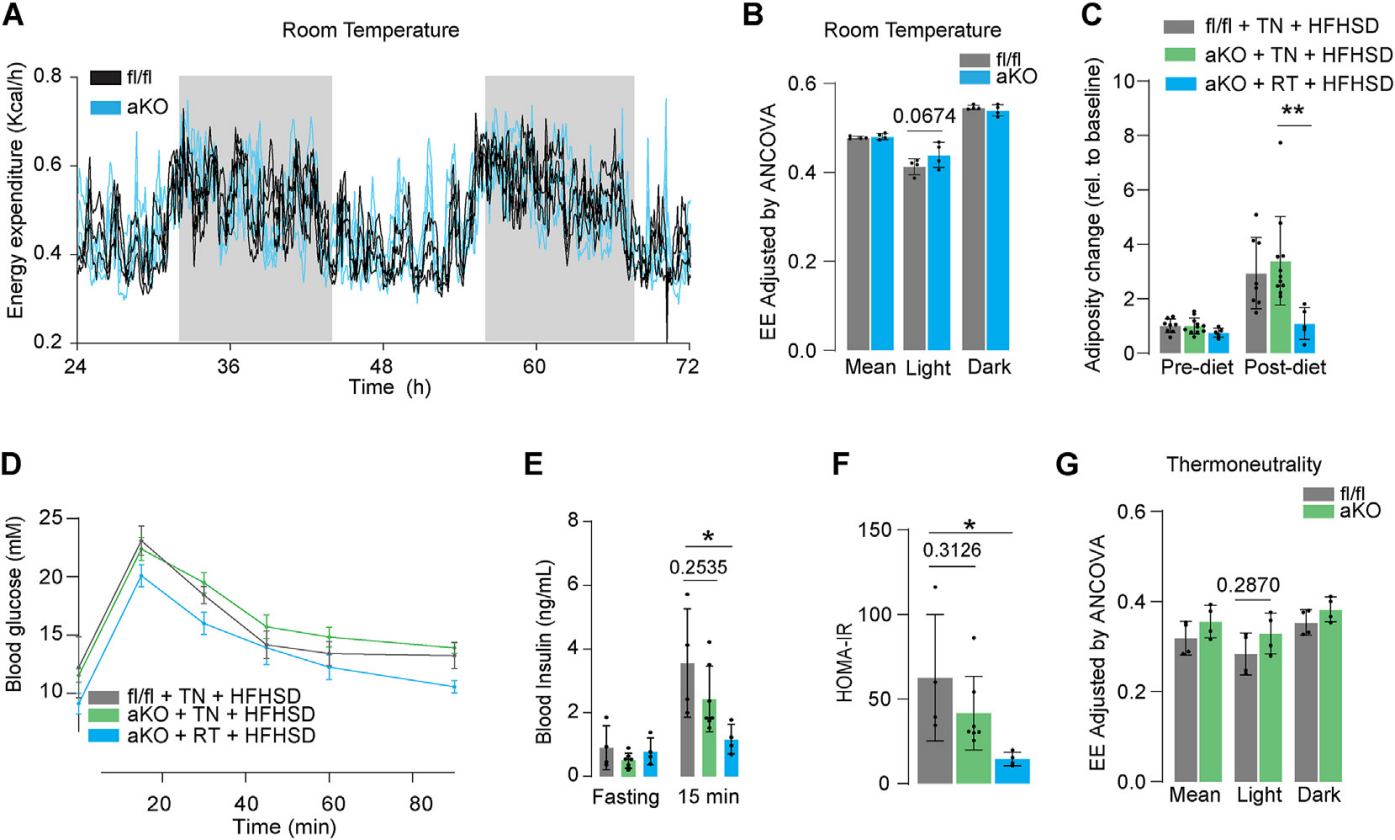
T**hermoneutrality reverses the protective phenotype observed in miPEP deficient mice**. (**A**) Raw energy expenditure (Kcal/h) for adipo-miPEP-KO (aKO, light blue) and miPEP^fl/fl^ (black) mice housed at room temperature over 48 h period (**B**) Quantification of energy expenditure ANCOVA adjusted during light and dark circles every 24 h Mean ± S.D., N = 4. p-value reported (**C**) Change in adiposity in miPEP^fl/fl^ (fl/fl, grey) and adipo-miPEP-KO mice (aKO, light green) in response to a high fat, high sugar diet (HFHSD) in thermoneutrality (TN) or room temperature (RT) relative to baseline. Mean ± S.D., N = 4–7, ∗p < 0.05, ∗∗∗p < 0.001 vs miPEP^fl/fl^ mice. (**D**) Oral Glucose tolerance test, (**E**) Blood insulin in fasting and at 15 min during the oral glucose tolerance test and (**F**) HOMA-IR after 4 wks of HFHSD in thermoneutrality (TN) or age-matched mice housed at room temperature (RT). Mean ± S.D., N = 4–7. ∗p < 0.05. Mean ± S.D., N = 4. p-value reported. (**G**) Mice were housed at thermoneutrality (TN) and fed a HFHSD for 4 weeks. Following this period, their EE was measured over 48 h under TN. EE was quantified (ANCOVA adjusted). Data are presented as mean ± S.D. (N = 4). The p-value is reported.

Given the observation that adipo-miPEP-KO mice were unable to expand their adipose tissue even when fed a HFHSD and despite consuming more food than control mice, we next explored energy expenditure (EE) in these mice. There was no significant difference in physical activity or respiratory exchange ratio (RER) in miPEP KO mice, (Sup. Figure 3A–D). Although adipo-miPEP-KO mice exhibited a small but consistent increase in EE during the light cycle; this effect did not reach statistical significance (Figure 4A,B).

The impact of just a small increment in EE on adiposity over a prolonged period is predicted to be quite significant [39]. It is important to consider that the sample size required to detect a significant difference of 3–5% in EE with sufficient power is ∼200 mice/genotypes [40]. To overcome the need for such a study, we reasoned that if the phenotype observed in adipo-miPEP-KO mice was due to a change in EE associated with thermoregulation this effect would probably be lost if animals were housed at thermoneutrality, and such an experiment would require far fewer animals [41]. To test this, we housed miPEP deficient and miPEP^fl/fl^ mice in a thermoneutral environment (30 °C) plus a HFFHSD treatment. Consistent with our hypothesis, at thermoneutrality adipo-miPEP-KO mice expanded their adipose tissue (Figure 4C), developed glucose intolerance (Figure 4D), insulin resistance (Figure 4E & F) and showed reduced EE (Figure 4G, Sup. Figure 3I) comparable with control littermates. These results suggest that deletion of miPEP in adipocytes triggers reduced adiposity and increased insulin sensitivity at room temperature by causing an increase in thermogenesis.

### 3.5 Adipo-miPEP-KO mice do not exhibit adipose tissue browning

**Figure 5:**
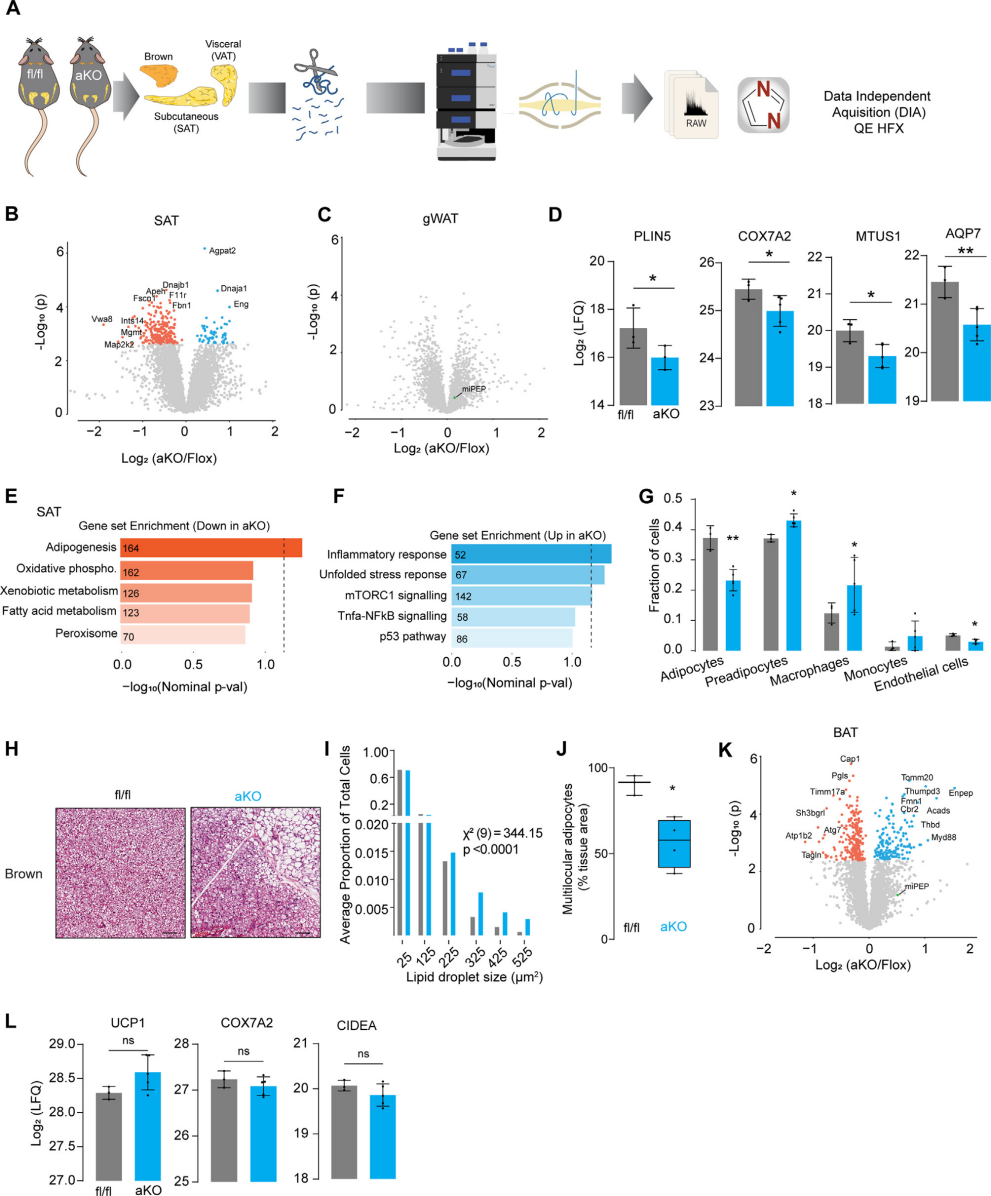
**adipo-miPEP-KO mice do not exhibit adipose tissue browning**. (**A**) Experimental workflow for global proteomics of gonadal white adipose tissue (gWAT), subcutaneous white adipose tissue (SAT) and brown adipose tissue (BAT) in aKO mice. (**B–C**) Volcano plot of relative protein abundance across adipose tissues. Orange, significatively downregulated proteins. Light blue, significatively upregulated proteins (−log10(p-val) = 2.2). (**D**) Abundance of proteins associated with browning, N = 3–5, Mean ± S.D. ∗p < 0.05 vs miPEP^fl/fl^. Gene set enrichment analysis of downregulated (**E**) and upregulated (**F**) proteins in adipo-miPEP-KO mice. Number of proteins associated with each pathway is shown within each bar. (**G**) Cell type deconvolution using proteomic data from SAT whole tissue aKO and miPEP^fl/fl^ (fl/fl) mice. Mean ± S.D., N = 3–5, ∗p < 0.05, ∗∗p < 0.01 vs miPEP^fl/fl^ mice. (**H**) Haematoxylin and eosin staining of brown adipose tissue (BAT). Scale bar 50 μm. (**I**) Quantification of the adipocyte size distribution for BAT (N = 5). (J) Percentage of cells with multilocular lipids droplets relative to the whole tissue area. N = 4, Median ± S.D. ∗p < 0.05 vs miPEP^fl/fl^. (**K**) Volcano plot of relative protein abundance in BAT. Orange, significatively downregulated proteins. Light blue, significatively upregulated proteins. (**L**) Abundance of proteins associated with BAT activity, N = 3–5, Mean ± S.D. ∗p < 0.05 vs miPEP^fl/fl^.

The fact that adipo-miPEP-KO are phenotypically normal in thermoneutral conditions suggests that increased metabolism, and consequently heat generation, occurs when these mice are housed at room temperature. One likely explanation for this is increased browning of white adipose tissue or increased activity of BAT, as these have been shown to play a major role in whole body thermogenesis in mice [42]. Since browning is generally accompanied by a predictable change in the adipose proteome, we conducted a proteomic analysis of SAT and gWAT in male mice (20 wks old) housed at room temperature with free access to NCD and water (Figure 5A).

Deletion of miPEP led to a tissue-specific remodelling of the proteome. For example, 59 proteins were upregulated and 241 proteins were downregulated in SAT (Padj < 0.05) (Figure 5B). Intriguingly, we did not observe any significant change in the proteome in gWAT from miPEP KO mice (Figure 5C). This is likely because adipocytes only constitute around 30% of the total cell population in the tissue and mitochondria are far less enriched in gWAT compared to SAT [14]. Consistent with this explanation, we did not observe a significant decrease in miPEP levels in gWAT from KO Mice whereas in isolated adipocytes miPEP was absent (Figure 2A). Surprisingly, the expression of key markers of browning like PLIN5, COX7A2, MTUS1, AQP7 [43] were reduced in SAT from miPEP KO mice (Figure 5D) suggesting that deletion of miPEP does not promote adipocyte browning.

To better understand the effect of miPEP deletion on the adipose tissue proteome we performed Gene Set enrichment analysis (GSEA) [34,44]. This revealed that adipogenesis and mitochondria were the major downregulated pathways (Figure 5E) and the inflammatory response was the most upregulated pathway in SAT (Figure 5F) suggesting that miPEP deletion has a profound effect on the cellular composition of SAT. To test this, we performed a cell type deconvolution of SAT utilising an adipose tissue single nuclei transcriptome database [35,36]. This analysis revealed that the proportion of adipocytes in SAT was reduced in adipo-miPEP-KO mice concomitant with an increased proportion of macrophages and preadipocytes (Figure 5H). Consistent with this observation, plasma proteomics of fasted male mice in NCD revealed that the levels of adipocyte-specific derived hormones such as adiponectin and adipsin were lower in adipo-miPEP-KO-mice (Sup. Figure 3F–H). Collectively these data suggest that miPEP deletion depletes SAT of mature adipocytes and increases the relative abundance of macrophages and preadipocytes.

Finally, because BAT is one of the major controllers of whole body metabolism [42] we tested if the reduced adiposity, as observed in adipo-miPEP-KO mice, is driven by BAT activation. Surprisingly, histological analysis in mice housed at room temperature revealed that miPEP deletion led to a whitening of BAT [45] with many cells exhibiting large unilocular lipid droplets, instead of the classic multilocular lipid droplets (p < 0.0001) (Figure 5H–J). This is not consistent with increased thermogenic function of BAT [45]. To confirm this, we next performed proteomics analysis on BAT. This revealed 397 differentially regulated proteins (P adj < 0.05) with 232 downregulated and 165 upregulated proteins (Figure 5K). GSEA revealed downregulation of proteins involved in mitochondria respiration. This suggests that loss of miPEP in brown adipocytes results in a loss of mitochondria likely due to the critical role of miPEP in mitochondrial proteostasis [18]. Similar to SAT, inflammatory related processes were upregulated in BAT (Sup. Figure 4A,B) suggesting that miPEP deletion leads to BAT dysfunction. Most notably, there was no significant increase in the expression of BAT specific proteins like UCP1, COX7A2 or CIDEA (Figure 5L) suggesting that miPEP deletion does not increase BAT activity.

### 3.6 Adipocyte specific miPEP-deletion increases skeletal muscle metabolism

In addition to BAT, skeletal muscle also contributes to adaptive thermoregulation via the activation of various metabolic futile cycles that consume ATP such as shivering, Ca^2+^ cycling, and mitochondrial uncoupling [46,47]. To test the potential role of skeletal muscle we next examined energy metabolism in various tissues.

**Figure 6:**
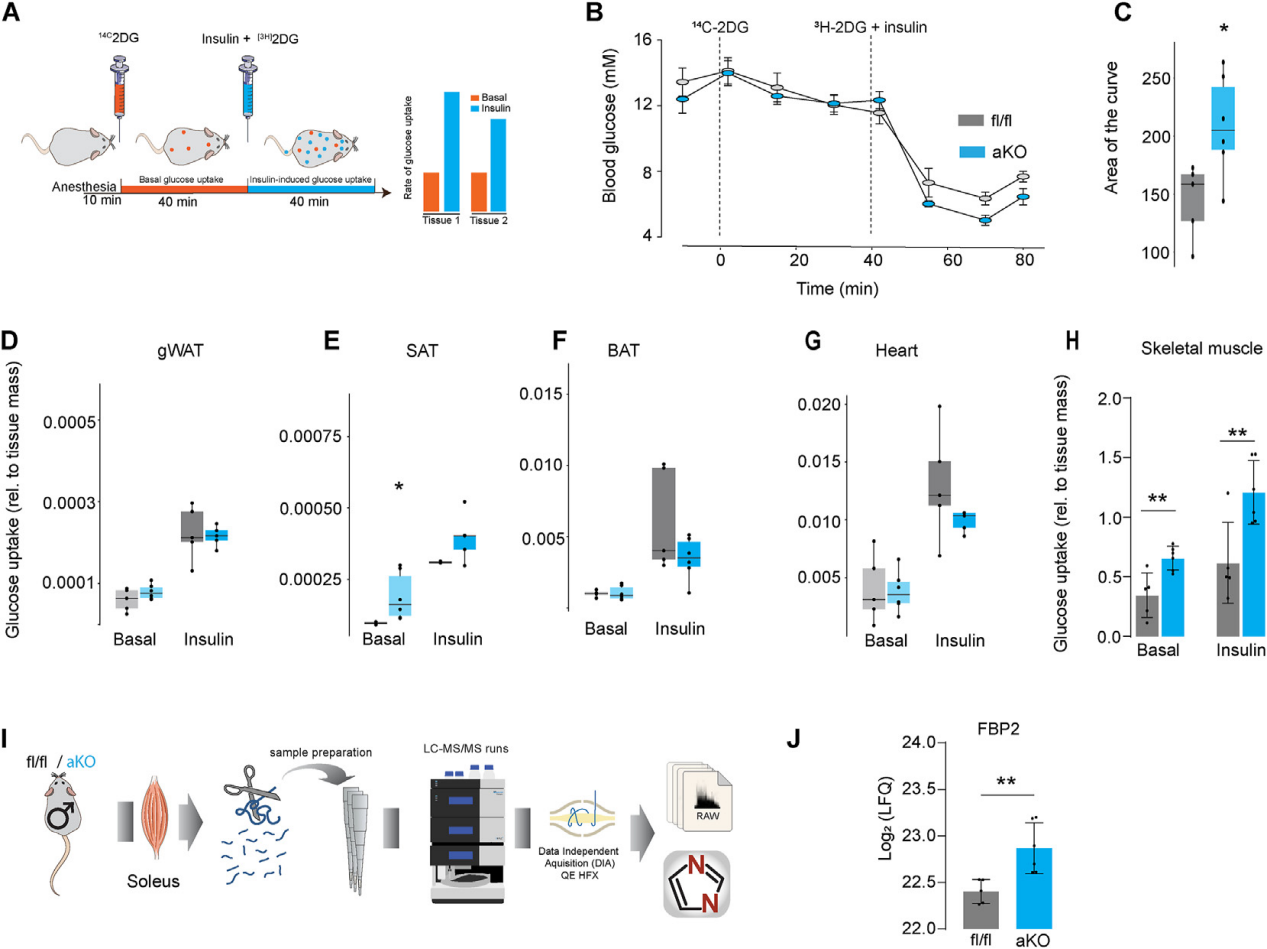
**Deletion of miPEP increases muscle metabolism**. (**A**) Experimental summary of the Dual Tracer Test (DTT) workflow and hypothetical data. (**B**) Blood glucose levels during the DTT. (**C**) Area of the curve after insulin administration in control (miPEP^fl/fl^, fl/fl) and adipocyte specific miPEP KO (aKO, light blue) mice. (**D–H**) Accumulation of basal [14C]2DG-6P and insulin-stimulated [3H]2DG-6P in Gonadal white adipose tissue (gWAT) (**D**), Subcutaneous adipose tissue (SAT) (**E**), Brown adipose tissue (BAT) (**F**), Heart (**G**), and skeletal muscle (**H**) was determined across genotypes. N = 5–6, ∗p < 0.05, ∗∗p < 0.01 vs miPEP^fl/fl^ mice. (**I**) Experimental workflow for global proteomics of soleus muscle in miPEP^fl/fl^ and aKO mice. (**J**) Abundance of Fbp2 in soleus, N = 5–6 Mean ± S.D. ∗∗p < 0.05 vs miPEP^fl/fl^.

Since cellular energy production is closely tied to glucose uptake and utilisation, employing glucose tracers like 2-deoxyglucose (2DG) offers a feasible approach to monitor metabolic activity [8,48]. In fact, 2DG is used to diagnose the presence of brown adipose tissue in humans due to this principle [49]. By studying glucose metabolism in the presence of insulin this also enables a much more accurate analysis of total energy metabolism as insulin blocks the supply of free fatty acids to the tissue [19]. Consequently, muscle and brown fat switch to glucose utilisation [19] and so this serves as a very sensitive index of total energy metabolism. Hence, we measured tissue-specific 2DG uptake in both adipo-miPEP-KO and miPEP^fl/fl^ littermates exposed to a HFHSD for 8 wks. We conducted our recently described dual tracer test (DTT) [27], which involves administering ^14C^2DG alone via retro-orbital injection, followed 40 min later by co-injection of ^3H^2DG together with a maximal insulin dose. This method enables measurement of both basal and insulin-stimulated 2DG uptake in the same tissue (Figure 6A). Blood glucose levels during DTT are shown in (Figure 6B). Injection of insulin elicited a significant reduction in the blood glucose concentration and led to more rapid disappearance of the insulin-stimulated tracer (^3H^2DG) than observed under basal conditions (Sup. Figure 4), consistent with an increased rate of 2DG uptake by peripheral tissues in response to insulin. In line with our previous observations in chow fed mice, adipo-miPEP-KO mice exhibited elevated insulin sensitivity (Figure 6B,C). Intriguingly, we did not observe any significant difference in insulin-induced glucose uptake in gWAT, BAT or heart from miPEP mice, whereas SAT had a small but significant increment in basal glucose uptake compared with miPEP^fl/f^ mice (Figure 6D,G). Interestingly, skeletal muscle showed a two-fold elevation in both basal and insulin-induced 2DG uptake (Figure 6H) consistent with an increase in muscle metabolism. These data suggest that increased metabolic activity by skeletal muscle might be the most likely contributor to the reduction in adiposity in adipo-miPEP-KO mice.

To determine if this increase in muscle metabolism might be accompanied by a change in proteins known to contribute to muscle thermogenesis, we next studied the muscle proteome. We conducted unbiased proteomics on soleus muscle samples from adipo-miPEP-KO and fl/fl littermates housed at room temperature (Figure 6I). As we were specifically interested in proteins that contribute to muscle thermogenesis, we specifically interrogated these proteins in our proteomes. We focused on proteins involved in ATP futile cycling as they have been broadly associated with muscle metabolism in response to cold [50]. This includes the mitochondrial ADP/ATP carrier (ANT1), the sarcoendoplasmic reticulum calcium pump (SERCA1), the ryanodine receptor calcium channel (RyR1), the SERCA regulatory protein sarcolipin (SLN), and the gluconeogenic proteins, fructose-bisphosphatase 2 (FBP2), as they have also been purported to form energy wasting futile cycles [46,[51], [53]]. Strikingly, adipocyte specific miPEP deletion resulted in a highly selective upregulation (p-val < 0.01, Log 2FC > 0.32) of FBP2 (Figure 6J). Notably, FBP2 overexpression is necessary for muscle adaptation to cold and it has previously been reported to enhance both basal and insulin-induced glucose levels in skeletal muscle [54], suggesting that FBP2 may play a causal role in mediating the increment in muscle metabolism observed in adipo-miPEP-KO mice.

## 4 Discussion

Mitochondria play a critical role in maintaining adipose tissue function and whole-body homoeostasis [55]. In the present study we unravel a novel function for adipocyte mitochondria whereby deletion of a mitochondrial peptidase results in a decline in adipocyte function that in turn leads to the increase in skeletal muscle metabolism likely involving the gluconeogenic protein FBP2. This program is sufficient to protect the mice against diet-induced obesity and insulin resistance.

Metabolic processes coupled with heat generation are now of considerable interest in terms of its potential anti-obesity effects [56]. Two primary processes are shivering and non-shivering thermogenesis. Shivering thermogenesis occurs when skeletal muscles contract involuntarily in response to cold stimuli, generating heat as a by-product of ATP hydrolysis [57]. Similarly non-shivering thermogenesis involves activation of ATP consumption cycles aside from shivering with consequent heat generation [57]. There are several non-shivering thermogenic pathways in mice and some of these are thought to extend to humans [58]. The most notorious involves upregulation of the uncoupling protein UCP1 in brown or beige adipocytes [59]. This protein transports protons across the inner mitochondrial membrane, uncoupling mitochondrial respiration from ATP production [60]. As a result, it converts the proton electrochemical gradient into heat, which expends energy and ultimately reduces overall body fat [61]. Several stimuli have been shown to activate this pathway including beta adrenergic agonists and cold temperature [60]. Additionally, UCP1-independent mechanisms of non-shivering thermogenesis can also regulate whole-body energy homoeostasis and adiposity [46,52,56]. For instance, the Creatine-dependent ATP-consuming cycle induces energy dissipation in beige and brown fat cells, although this mechanism remains controversial [62,63]. Another example is the Ca^2+^ cycling, which is mediated by the calcium channel RYR2 and the calcium pump SERCA2b located in the endoplasmic reticulum, contributing to enhanced energy expenditure in beige fat [52]. Furthermore, the regulatory protein SLN facilitates uncoupling of ATP hydrolysis from Ca^2+^ transport in skeletal muscle [46]. Other thermogenic pathways, such as those involving thyroid hormones and lipid cycling, have been discussed extensively in other sources [50]. Due to the lack of signs of beiging in white adipose tissue and the presence of markers indicating dysfunction in BAT, we exclude the possibility of brown or beige adipocytes as drivers of the lean phenotype in adipo-miPEP-KO mice. Rather our metabolic analyses point to skeletal muscle as the major contributor of the adipo-miPEP-KO phenotype. Shivering-induced thermogenesis is unlikely for several reasons. First, shivering thermogenesis is a very rapid response whereas the thermogenic phenotype in miPEP required many weeks to emerge [64]. Moreover, *Ucp1*-deficient mice can adapt to cold only when the reduction in temperature is slow (over 2 wks) indicating that the shivering capacity of mice acutely exposed to cold cannot maintain sustained body temperature [59,65]. Thus, similar to adipo-miPEP-KO mice, *Ucp1*-deficient mice adapt to cold by activating non-shivering thermogenic mechanisms [65]. Second, increased shivering leads to alterations in muscle physiology resembling those seen with exercise training [66]. However, this was not evident from our proteomics analysis. This indicates that shivering thermogenesis is not likely the primary driver of the increase in metabolic activity of adipo-miPEP-KO mice.

Based on our results, muscle non-shivering thermogenesis emerges as a potential metabolic pathway in adipo-miPEP-KO mice. This is supported by our observation of increased glucose uptake and the upregulation of FBP2 levels specifically in the muscle of these mice. Interestingly, FBP2 was the only metabolic protein that showed a significant alteration in miPEP deficient animals. FBP2 is a gluconeogenic enzyme that controls the cycling between fructose-6-phosphate and fructose-1,6-bisphosphate which is critical for regulating whole body metabolism and thermal-stability [67]. Normally the rate of glycolysis in muscle vastly exceeds the rate of gluconeogenesis thus making any cycle involving simultaneous activation of these pathways highly unlikely [54]. However, even a small increase in FBP2 abundance is thought to elevate ATP-turnover by up to 10–20%, which has a significant impact on heat generation and whole body metabolism [54]. Induction of the gluconeogenic enzyme FBP2 has been shown to occur with exercise training and cold challenge [68] and under these circumstances induced ATP cycling and heat generation has been reported [53]. Very recently overexpression of FBP2 in muscle was shown to be sufficient to increase muscle glucose metabolism [54] analogous to that seen in adipo-miPEP-KO mice (Figure 6). Future studies will be necessary to confirm this observation by crossing our miPEP mice with a muscle specific FBP2 KO mouse.

This model gives rise to several other questions. How is FBP2 upregulation in muscle mediated and how does miPEP deletion in adipose tissue interact with this pathway? It has been shown that FBP2 expression is under the control of the orphan nuclear receptor Nur77 [69,70]. Although Nur77 ligands have not yet been identified, recent evidence indicates that fatty acid metabolites may have the ability to activate Nur77 [71]. Hence, analyses of these or related molecules in miPEP KO is warranted. It is likely that such molecules/ligands arise from adipocytes as this is where miPEP was deleted. Our data suggest that deletion of miPEP, especially in adipocytes that are rich in mitochondria such as brown and subcutaneous adipocytes, results in cellular stress, as evidenced by the upregulation of inflammatory pathways. Therefore, it is plausible that this stress pathway is involved in the generation of a factor that activates the FBP2 cycle in muscle. The hormone FGF21 is among the most studied factors secreted by cells under mitochondrial stress [[72], [73], [74]]. FGF21 is known to enhance whole body metabolism by inducing browning of SAT, thereby providing protection against diet-induced obesity [74,75]. However, deletion of miPEP in adipocytes did not influence plasma levels of FGF21 (Sup. Figure 3E), indicating that the observed phenotype is driven by an FGF21-independent mechanism. Interestingly, the protein level of FBP2 is increased in the muscle of mice exposed to a HFHSD [76]. On the other hand, mice housed for 4 weeks at 4 °C (unpublished data) revealed no significant changes. This finding supports the hypothesis that the upregulation of FBP2 in adipo-miPEP-KO mice is driven by a specific signal rather than a general response to metabolic challenges. Exploring the pathway linking fat and muscle in future research will be exciting, and the adipo-miPEP-KO mice offer a promising avenue for such investigations.

The loss of miPEP in interscapular adipose tissue resulted in whitening of BAT, a condition characterised by the inability of BAT to maintain body temperature [45]. This white-like phenotype of BAT has been observed in several mouse models deficient in mitochondrial proteins such as *Cpt2* [77], *Cox7rp* [78], *Clpp* [79] and *Ucp1* [65] suggesting that mitochondrial function is crucial for BAT function. Because room temperature acts as a cold stress for mice, it triggers BAT metabolism leading to increased mitochondrial protein content [80]. Given miPEP's crucial role in protein maturation [[81], [82], [83]], it is likely that miPEP absence impedes the maturation of newly imported mitochondrial proteins, affecting mitochondrial function, blocking BAT function. This aligns with the observed downregulation of respiratory-related proteins in BAT of miPEP-deficient mice. We speculate that deleting miPEP in adipocytes activates alternative thermogenic routes that are less efficient in cold defence. The increase in muscle metabolism, coupled with the lower efficiency in heat generation per gram of nutrient oxidised, could collectively account for the leaner phenotype observed in miPEP-deficient mice. Our results suggest that this defect in BAT is driving the inability to induce thermogenesis in this tissue, which in turn triggers a compensatory increase in muscle mediated thermogenesis.

In summary, our study identifies a unique tissue cross talk between adipose tissue and skeletal muscle leading to the induction of the levels of FBP2 which is predicted to drive an increment in muscle metabolism that results in reduced adiposity. While further research is required to elucidate the detailed molecular wiring of this pathway this is quite exciting as it suggests that while there is currently much focus on adipose tissue as a metabolic driver, skeletal muscle still represents a viable and safe pathway that could have important therapeutic efficacy.

## Lead contact

Further information and requests for resources and reagents should be directed to and will be fulfilled by the Lead Contacts, Alexis Diaz-Vegas (alexis.diaz@sydney.edu.au) and David James (David.james@sydney.edu.au).

## CRediT authorship contribution statement

**Alexis Diaz-Vegas:** Writing – review & editing, Writing – original draft, Visualization, Project administration, Methodology, Investigation, Funding acquisition, Formal analysis, Data curation, Conceptualization. **Kristen C. Cooke:** Writing – review & editing, Resources, Methodology, Investigation. **Harry B. Cutler:** Writing – review & editing, Visualization, Methodology, Investigation, Formal analysis, Conceptualization. **Belinda Yau:** Writing – review & editing, Investigation, Formal analysis. **Stewart W.C. Masson:** Writing – review & editing, Investigation, Formal analysis, Conceptualization. **Dylan Harney:** Writing – review & editing, Methodology, Investigation. **Oliver K. Fuller:** Writing – review & editing, Investigation, Formal analysis, Data curation. **Meg Potter:** Writing – review & editing, Methodology. **Søren Madsen:** Writing – review & editing, Formal analysis. **Niamh R. Craw:** Writing – review & editing, Methodology, Investigation. **Yiju Zhang:** Writing – original draft, Investigation. **Cesar L. Moreno:** Writing – original draft, Methodology, Investigation. **Melkam Kebede:** Writing – original draft, Investigation. **G. Gregory Neely:** Writing – review & editing, Methodology, Investigation. **Jacqueline Stöckli:** Writing – review & editing, Methodology, Investigation. **James G. Burchfield:** Writing – review & editing, Investigation, Funding acquisition. **David E. James:** Writing – review & editing, Writing – original draft, Supervision, Project administration, Funding acquisition, Conceptualization.

## Declaration of competing interest

The authors declare that there are no competing financial interests that could have influenced the work reported in this paper.

## Data Availability

Data will be made available on request.

## Appendix A Supplementary data

Supplementary Figure 1 **Additional characterisation of miPEP deficient mice in normal chow diet** (A) Glucose induced insulin secretion in isolated pancreatic islets (B) Total insulin content in isolated pancreatic islets. Mean ± S.D., N = 6–7. Temporal change in body weight (C) and lean mass (D) for miPEP^fl/fl^ (fl/fl) and adipo-miPEP-KO mice (aKO) are shown N = 4–5 Mean ± S.D. (E) Adiposity in control miPEP^fl/fl^ (fl/fl) and adipo-miPEP-KO (aKO) and age matched Wild type (WT) mice at 16 wks old. Mean ± S.D., N = 4–10, ∗∗∗p < 0.001, ∗∗∗∗p < 0.0001 as indicated. (F) Weight of fat depots in miPEP^fl/fl^ (fl/fl, grey) and miPEP deficient (aKO, light blue) mice. Mean ± S.D., N = 3–7, ∗p < 0.05, ∗∗p < 0.01, ∗∗∗p < 0.001 as indicated. Supplementary Figure 1

Supplementary Figure 2 **miPEP deficient female mice are protected against diet induced obesity and glucose intolerance**. (A) Experimental workflow HFHSD = High fat, high sugar diet. OGTT = Oral glucose tolerance test, LTT = Lipid Tolerance Test. Changes in (B) body weight , (C) fat mass and (D) adiposity pre-diet, at 2.5 wks and 8 wks of diet is shown. Mean ± S.D., N = 9–11, ∗∗p < 0.01, ∗∗∗p < 0.001, ∗∗∗∗p < 0.0001. (E) Representative dual energy X-ray absorptiometry (DEXA) image for miPEP^fl/fl^ (fl/fl) and adipo-miPEP-KO (aKO) after 8 wks of diet intervention. (F) Food intake Mean ± S.D., N = 3, ∗∗p < 0.01 vs miPEP^fl/fl^ (G) Fasting triglycerides Mean ± S.D., N = 5–6. (H) Kinetic and (I) area of the curve of lipid tolerance test using oral gavage of intralipids. Mean ± S.D., N = 5–6. After 8 wks of HFHSD (J) Oral Glucose tolerance test, (K) Fasting glucose was determined. (L) Blood insulin in fasting and at 15 min during the Oral Glucose tolerance test. (M) HOMA-IR (N) Matsuda Index. Mean ± S.D., N = 7–10, ∗p < 0.05, ∗∗p < 0.01 vs miPEP^fl/fl^ mice. Supplementary Figure 2

Supplementary Figure 3 **miPEP deletion does not affect RER, food intake or locomotor activity when fed normal chow diet**. (A) Respiratory respiratory exchange ratio (RER) for miPEP KO (aKO, light blue) and miPEP^fl/fl^ (fl/fl, black) mice over 48 h period (B) Quantification of RER during light and dark circles as well as the average RER every 24 h. Mean ± S.D., N = 8. (C) Food intake over 48 h Mean ± S.D., N = 6 (D) Locomotor activity over 48 h. Mean ± S.D., N = 3–4. (E) plasma levels of Fgf21. Mean ± S.D., N = 4–5 (F) Volcano plot of relative protein abundance in plasma. (G–H) Levels of proteins associated with adipose tissue abundance, N = 3–5, Mean ± S.D. ∗p < 0.05 vs miPEP^fl/fl^. (I) Raw energy expenditure (Kcal/h) for adipo-miPEP-KO (aKO, light green) and miPEP^fl/fl^ (black) mice housed in thermoneutrality plus HDHSD. N = 4, Mean ± S.D. Supplementary Figure 3

Supplementary Figure 4 **Gene set enrichment analysis of brown adipose tissue**. (A) Gene set enrichment analysis of downregulated (A) and upregulated (B) proteins in KO mice. Number of proteins associated with each pathway is shown within each bar. Supplementary Figure 4

Supplementary Figure 5 **Minimal data Dual Tracer Test (DTT)** (A) Radioactivity in blood (DPM) measured during the DTT. (B) Decay constant in basal and after insulin administration. Glucose uptake in basal and after insulin stimulation in soleus (C) and Quadriceps (D) is shown. (E) Lean and fat mass at the end of the intervention is shown. Mean ± S.D., N = 5–6, ∗p < 0.05, ∗∗p < 0.01 as denoted. (F) Volcano plot of relative protein abundance in plasma. Orange for downregulated and Blue for upregulated proteins. (L) Abundance of proteins associated with muscle thermogenesis. Supplementary Figure 5
