# Supplementary material for: Deletion of miPEP in adipocytes protects against obesity and insulin resistance by boosting muscle metabolism
Source: Mol Metab. 2024 Jul 1;86:101983. doi: 10.1016/j.molmet.2024.101983 (PMC11292358; doi:10.1016/j.molmet.2024.101983)

Supplementary Figure 1. Additional characterisation of miPEP deficient mice in normal chow diet

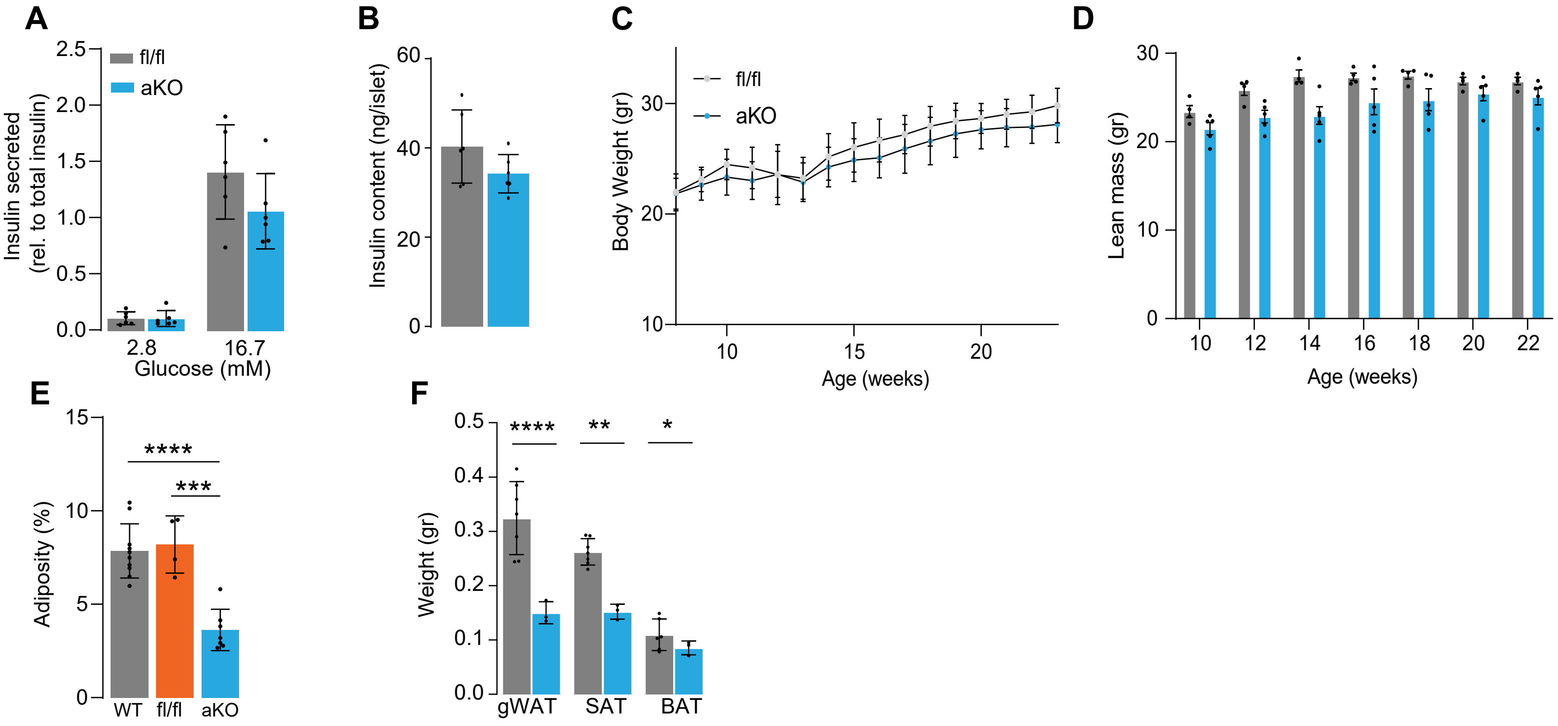

Supplement: Supplementary Figure 1 — Additional characterisation of miPEP deficient mice in normal chow diet (A) Glucose induced insulin secretion in isolated pancreatic islets (B) Total insulin content in isolated pancreatic islets. Mean ± S.D., N = 6–7. Temporal change in body weight (C) and lean mass (D) for miPEPfl/fl (fl/fl) and adipo-miPEP-KO mice (aKO) are shown N = 4–5 Mean ± S.D. (E) Adiposity in control miPEPfl/fl (fl/fl) and adipo-miPEP-KO (aKO) and age matched Wild type (WT) mice at 16 wks old. Mean ± S.D., N = 4–10, ∗∗∗p < 0.001, ∗∗∗∗p < 0.0001 as indicated. (F) Weight of fat depots in miPEPfl/fl (fl/fl, grey) and miPEP deficient (aKO, light blue) mice. Mean ± S.D., N = 3–7, ∗p < 0.05, ∗∗p < 0.01, ∗∗∗p < 0.001 as indicated. [file mmc1.pdf]
