# Supplementary material for: Deletion of miPEP in adipocytes protects against obesity and insulin resistance by boosting muscle metabolism
Source: Mol Metab. 2024 Jul 1;86:101983. doi: 10.1016/j.molmet.2024.101983 (PMC11292358; doi:10.1016/j.molmet.2024.101983)

Supplementary Figure 2. miPEP deficient female mice are protected against diet induced obesity and glucose intolerance.

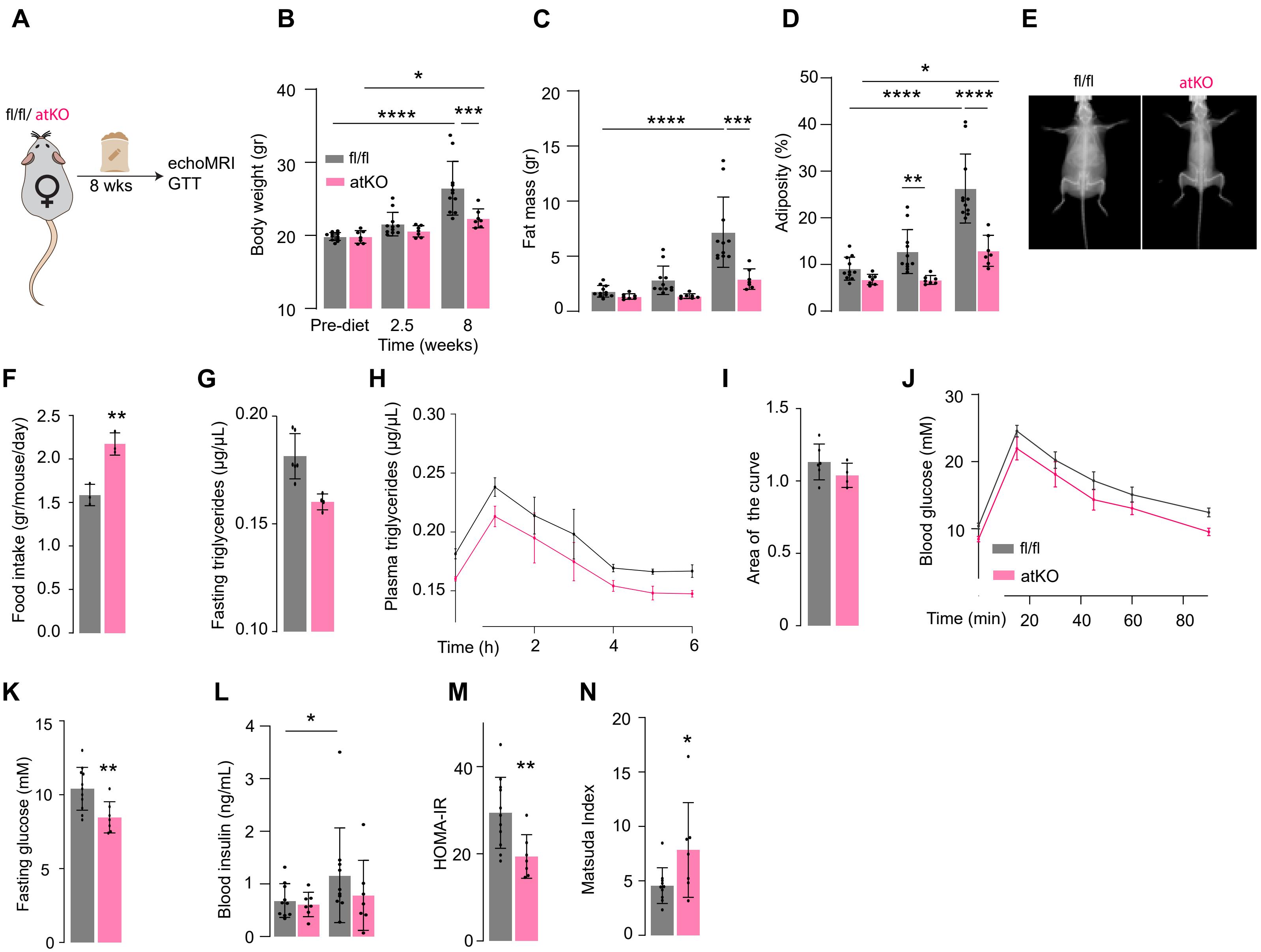

Supplement: Supplementary Figure 2 — miPEP deficient female mice are protected against diet induced obesity and glucose intolerance. (A) Experimental workflow HFHSD = High fat, high sugar diet. OGTT = Oral glucose tolerance test, LTT = Lipid Tolerance Test. Changes in (B) body weight , (C) fat mass and (D) adiposity pre-diet, at 2.5 wks and 8 wks of diet is shown. Mean ± S.D., N = 9–11, ∗∗p < 0.01, ∗∗∗p < 0.001, ∗∗∗∗p < 0.0001. (E) Representative dual energy X-ray absorptiometry (DEXA) image for miPEPfl/fl (fl/fl) and adipo-miPEP-KO (aKO) after 8 wks of diet intervention. (F) Food intake Mean ± S.D., N = 3, ∗∗p < 0.01 vs miPEPfl/fl (G) Fasting triglycerides Mean ± S.D., N = 5–6. (H) Kinetic and (I) area of the curve of lipid tolerance test using oral gavage of intralipids. Mean ± S.D., N = 5–6. After 8 wks of HFHSD (J) Oral Glucose tolerance test, (K) Fasting glucose was determined. (L) Blood insulin in fasting and at 15 min during the Oral Glucose tolerance test. (M) HOMA-IR (N) Matsuda Index. Mean ± S.D., N = 7–10, ∗p < 0.05, ∗∗p < 0.01 vs miPEPfl/fl mice. [file mmc2.pdf]
