# Supplementary material for: Deletion of miPEP in adipocytes protects against obesity and insulin resistance by boosting muscle metabolism
Source: Mol Metab. 2024 Jul 1;86:101983. doi: 10.1016/j.molmet.2024.101983 (PMC11292358; doi:10.1016/j.molmet.2024.101983)

Supplementary Figure 3. miPEP deletion does not affect RER, food intake or locomotor activity when fed normal chow diet.

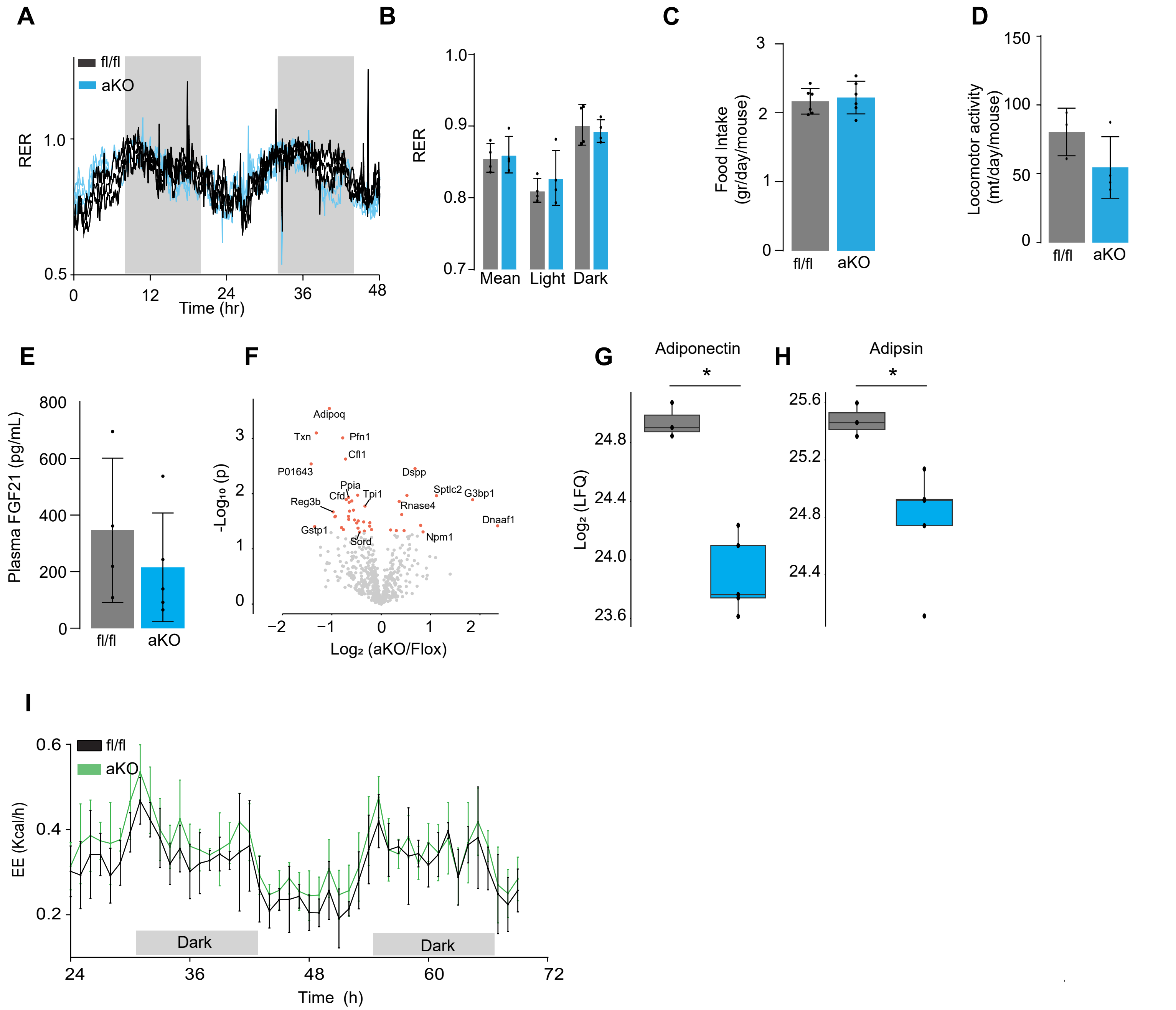

Supplement: Supplementary Figure 3 — miPEP deletion does not affect RER, food intake or locomotor activity when fed normal chow diet. (A) Respiratory respiratory exchange ratio (RER) for miPEP KO (aKO, light blue) and miPEPfl/fl (fl/fl, black) mice over 48 h period (B) Quantification of RER during light and dark circles as well as the average RER every 24 h. Mean ± S.D., N = 8. (C) Food intake over 48 h Mean ± S.D., N = 6 (D) Locomotor activity over 48 h. Mean ± S.D., N = 3–4. (E) plasma levels of Fgf21. Mean ± S.D., N = 4–5 (F) Volcano plot of relative protein abundance in plasma. (G–H) Levels of proteins associated with adipose tissue abundance, N = 3–5, Mean ± S.D. ∗p < 0.05 vs miPEPfl/fl. (I) Raw energy expenditure (Kcal/h) for adipo-miPEP-KO (aKO, light green) and miPEPfl/fl (black) mice housed in thermoneutrality plus HDHSD. N = 4, Mean ± S.D. [file mmc3.pdf]
