# Supplementary figures and images for: Deletion of miPEP in adipocytes protects against obesity and insulin resistance by boosting muscle metabolism
Source: Mol Metab. 2024 Jul 1;86:101983. doi: 10.1016/j.molmet.2024.101983 (PMC11292358; doi:10.1016/j.molmet.2024.101983)

Supplementary Figure 4. Gene set enrichment analysis of brown adipose tissue.

A

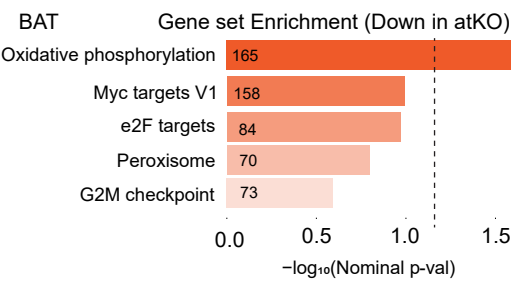

B

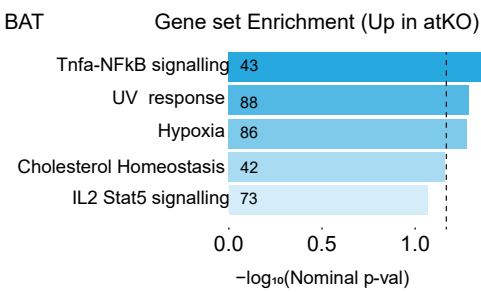

Supplement: Supplementary Figure 4 — Gene set enrichment analysis of brown adipose tissue. (A) Gene set enrichment analysis of downregulated (A) and upregulated (B) proteins in KO mice. Number of proteins associated with each pathway is shown within each bar. [file mmc4.pdf]

Supplementary Figure 5. Minimal data Dual Tracer Test (DTT)

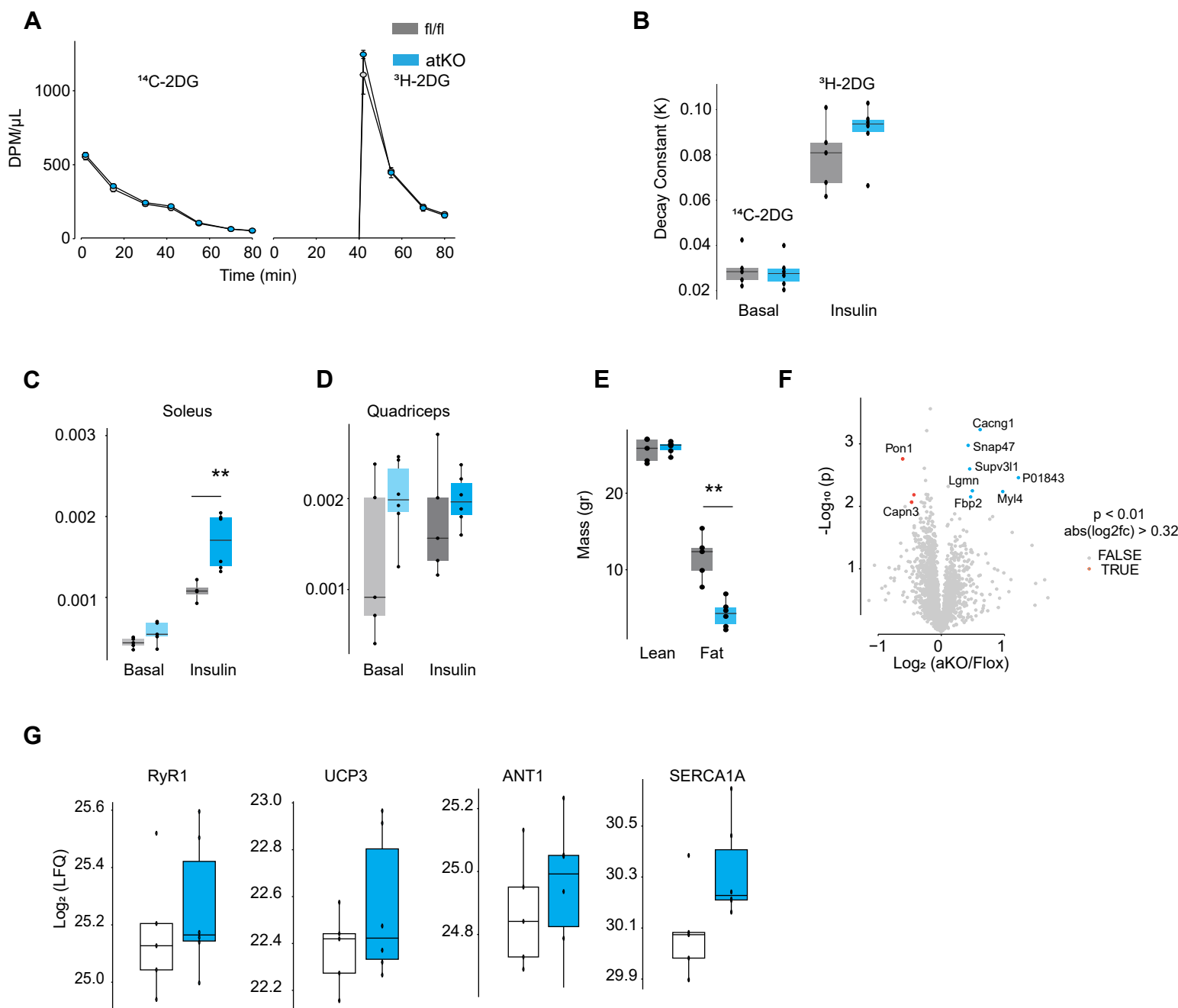

Supplement: Supplementary Figure 5 — Minimal data Dual Tracer Test (DTT) (A) Radioactivity in blood (DPM) measured during the DTT. (B) Decay constant in basal and after insulin administration. Glucose uptake in basal and after insulin stimulation in soleus (C) and Quadriceps (D) is shown. (E) Lean and fat mass at the end of the intervention is shown. Mean ± S.D., N = 5–6, ∗p < 0.05, ∗∗p < 0.01 as denoted. (F) Volcano plot of relative protein abundance in plasma. Orange for downregulated and Blue for upregulated proteins. (L) Abundance of proteins associated with muscle thermogenesis. [file mmc5.pdf]
